# Synthesis, In Vitro Biological Evaluation, and Oxidative Transformation of New Flavonol Derivatives: The Possible Role of the Phenyl-*N*,*N*-Dimethylamino Group

**DOI:** 10.3390/molecules23123161

**Published:** 2018-11-30

**Authors:** Peter Szabados-Furjesi, David Pajtas, Aliz Barta, Evelin Csepanyi, Attila Kiss-Szikszai, Arpad Tosaki, Istvan Bak

**Affiliations:** 1 Department of Bioanalytical Chemistry, Faculty of Pharmacy, University of Debrecen, Debrecen H-4032, Hungary; szabados-furjesi.peter@pharm.unideb.hu (P.S.-F.); csepanyi.evelin@pharm.unideb.hu (E.C.); 2 Department of Biophysics and Cell Biology, Faculty of Medicine, University of Debrecen, Debrecen H-4032, Hungary; pajtas.david@med.unideb.hu (D.P.); barta.aliz@med.unideb.hu (A.B.); 3 Department of Organic Chemistry, Faculty of Science and Technology, University of Debrecen, Debrecen H-4032, Hungary; kiss.attila@science.unideb.hu; 4 Department of Pharmacology, Faculty of Pharmacy, University of Debrecen, Debrecen H-4032, Hungary; tosaki.arpad@pharm.unideb.hu

**Keywords:** antioxidant, flavonoid, flavonol, cytotoxicity, oxidative metabolism

## Abstract

Six new flavonols (**6a**–**f**) were synthesized with Claisen–Schmidt and Suzuki reactions and they were fully characterized by spectroscopic methods. In order to evaluate their antioxidant activities, their oxygen radical absorption capacity and ferric reducing antioxidant power were measured, along with their free radical scavenging activity against 2,2′-azino-bis(3-ethylbenzothiazoline-6-sulphonic acid) and 2,2-diphenyl-1-picrylhydrazylradicals. In addition, their cytotoxicity on H9c2 cardiomyoblast cells was also assessed by a 3-(4,5-dimethylthiazol-2-yl)-2,5-diphenyltetrazolium bromide assay. Compounds bearing the phenyl-*N*,*N*-dimethylamino group (**6a**, **6c**, and **6e**) exhibited promising antioxidant potency and did not have any cytotoxic effect. After a consideration of these data, the oxidative transformation of the **6c** compound was investigated in vitro with a chemical Fenton reaction and the identification of the formed oxidation products was performed by mass spectrometry. Two potential metabolites were detected. Based on these results, compound **6c** can be a model compound for future developments. Overall, this work has proved the involvement of the phenyl-*N*,*N*-dimethylamino group in the antioxidant activity of flavonols.

## 1. Introduction

During oxidative/nitrosative stress, there is a lack of balance between the endogenous antioxidant system and the prooxidants, which could result in the excess formation of the latter. Reactive oxygen (ROS) and nitrogen species (RNS) can irreversibly damage lipids, proteins, nucleic acids, and other biomacromolecules contributing to the development and progression of several diseases, for instance, attention deficit hyperactivity disorder [1], cancer [2], Parkinson’s disease [3], Alzheimer’s disease [4], atherosclerosis [5], heart failure [6], and myocardial infarction [7]. In order to inhibit lipid peroxidation and protect the cell membranes, proteins, and nucleic acids from the ROS- and RNS-caused damages, the balance must be restored. This can be achieved by providing exogenous antioxidants to act as an active contributors of the endogenous antioxidant defense system. The most frequently used exogenous antioxidants are ascorbic acid, vitamin E, carotenoids, and different polyphenolic compounds, including flavonoids. These exogenous antioxidants are mostly produced by plants, and possess promising biological effects [4,8,9,10]. The flavonoids have been studied thoroughly in the past decades; they possess a broad range of beneficial biological effects, such as anti-inflammatory, antiviral, antibacterial, and antiallergic activities [11,12]. In further studies, flavonoids were proved to be powerful antioxidants [13,14]. Based upon the structural complexity, flavonoids can be divided into six groups: anthocyanins, flavan-3-ols, flavanones, flavones, isoflavones, and flavonols, which are a 3-hydroxyflavone backboned subclass of flavonoids with a wide range of biological activity [15,16,17]. Furthermore, the authors of the present study have previously investigated the antioxidant effects of different flavonoid-type chromone derivatives [18] and found that the 4′-*N*,*N*-dimethylamino-flavon was the most active tested compound. Hence, the amino group promotes free-radical scavenger activity [19]; i.e., the introduction of differentially *N*-substituted amino groups into the molecule could modify the antioxidant activity of the different flavonoids [20,21,22]. On the other hand, *O*-methylation of flavonoids could result in higher metabolic stability, increased bioavailability, and better tissue distribution [23,24,25]; however, the methylation of hydroxyl groups could decrease the antioxidant activity [26]. Based on these results, the aim of the present work was to evaluate the antioxidant potential and cytotoxic activity of six, newly synthetized *O*-methylated flavonol derivatives (Table 1) and investigate the effect of phenyl-*N*,*N*-dimethylamino and methoxy groups on the antioxidant potency.

The tested compounds were synthetized by Claisen-Schmidt condensation followed by a ring closure. The side chains were built in with a Suzuki reaction. The radical scavenging activity was determined by 2,2-diphenyl-1-picrylhydrazyl (DPPH) and 2,2′-azino-bis(3-ethylbenzothiazoline-6-sulphonic acid) (ABTS) radical scavenging assays. Moreover, the oxygen radical absorption capacity (ORAC) and ferric reducing antioxidant power (FRAP) of the compounds were also measured. In further experiments, the cytotoxicity of the compounds was studied by 3-(4,5-dimethylthiazol-2-yl)-2,5-diphenyltetrazolium bromide (MTT) assay on H9c2 cardyomyoblastoma cell cultures. In addition, we investigated the oxidative transformation of the molecules by the chemical Fenton reaction as a biomimetic model system of biotransformation.

## 2. Results and Discussion

### 2.1. Chemistry

**Scheme 1:**
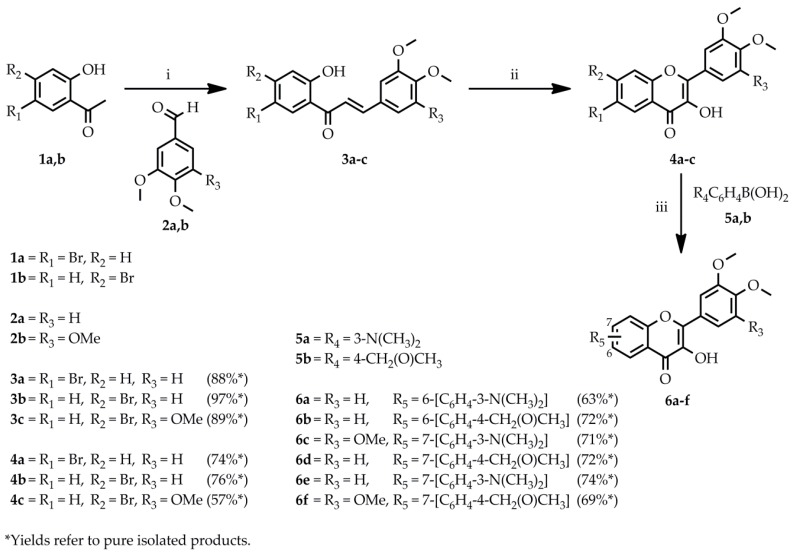
Synthesis of 6a-f. Reagents and reaction condition: (i) **1a**,**b**, benzaldehyde **2a**,**b** (1.05 equiv.), 50% aq. NaOH (4 equiv.), MeOH, room temperature, 24 h; (ii) **3a**–**c**, 8% aq. NaOH (3.1 equiv.), 30% H_2_O_2_ (14.1 equiv.), EtOH, room temperature, 2 h; (iii) **4a**–**c**, **5a**,**b** (2 equiv.), KF (3 equiv.), Pd(OAc)_2_ (5 mol%), XPhos (10 mol%), toluene/t-BuOH (6:1), argon atmosphere, 100 °C, 4 h.

Scheme 1 illustrates the preparation of the flavonol backbone (**4a**–**c**) used for the synthesis of the compounds of interest (**6a**–**f**).

As a first step, the Claisen-Schmidt condensation of the commercially available bromoacetophenones (**1a**,**b**) and benzaldehydes (**2a**,**b**) in MeOH provided the corresponding **3a**–**c** chalcones (88–97%) using 4 equiv. NaOH as a base. The cyclization of these chalcones (**3a**–**c**) into the **4a**–**c** key intermediates (57–76%) was performed using 14.1 equiv. 30% H_2_O_2_ in EtOH. Then, the Suzuki coupling of bromoflavonol derivatives (**4a**–**c**) with the appropriate boronic acid (**5a**,**b**) resulted in the desired **6a**–**f** flavonols (63–74%) using potassium-fluoride, Pd(OAc)_2_ as a palladium source, and XPhos as a ligand in toluene/t-BuOH (6:1) under argon atmosphere.

### 2.2. Antioxidant Activity

**Figure 1:**
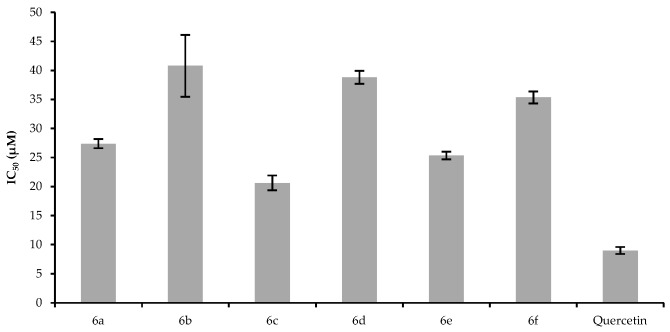
The IC**_50_** ABTS**^●+^** scavenging rate of the tested flavonol derivatives and quercetin standard. The inhibition percentage was determined at 120 min in the concentration range of 10–200 µM.

To determine the antioxidant properties of the target compounds, four assays were performed. During the ABTS assay, the ABTS radical cation scavenging ability was evaluated. The half-maximal scavenging rate (IC_50_) was calculated for each compound from the inhibition percentage at 120 min in the concentration range of 10–200 µM (Figure 1).

**Figure 2:**
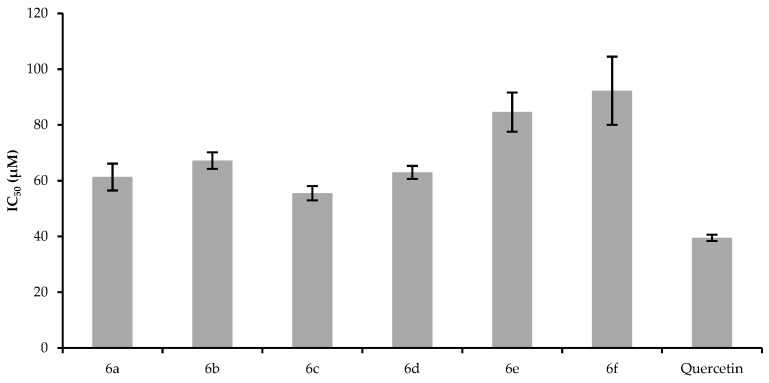
The IC_50_ DPPH scavenging rate of the tested flavonol derivatives and quercetin standard. The inhibition percentage was determined at 90 min in the concentration range of 10–200 µM.

The results show that compound **6c** demonstrated the highest scavenging potency by having the lowest IC_50_ value, followed by **6e** and **6a**, in the concentration range of 10–200 µM. By having the phenyl-*N*,*N*-dimethylamino group, these compounds (**6a**, **6c**, and **6e**) are better electron donors than compounds with methoxymethyl groups (**6b**, **6d**, and **6f**) because of the electronegativity difference. During the single electron-transfer process, the ABTS radical cation abstracts an electron and the flavonol may act as a donor and, thus, regenerates the original form and causes a detectable change in the absorbance at 737 nm. The greater the change in the absorbance, the higher the scavenging ability of the investigated compound. The principle of the DPPH assay is similar to that of the ABTS assay; i.e., DPPH is a stable radical and its solution is deep violet, since decolorization due to the reduction of the radical by the antioxidant can be detected at 515 nm by a spectrophotometer. Figure 2 depicts the calculated IC_50_ values for each compound from the inhibition percentage at 90 min in the concentration range of 10–200 µM.

**Figure 3:**
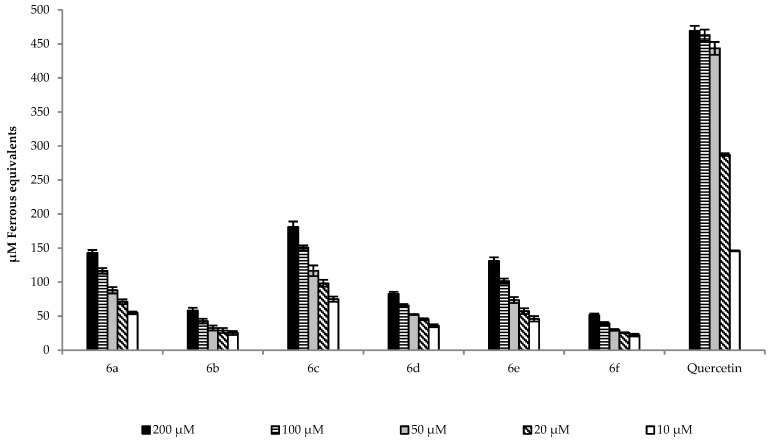
FRAP values of the investigated compounds. The fresh FRAP working solution consisted of acetate buffer, 2,4,6-tris(2-pyridyl)-s-triazine (TPTZ), and FeCl_3_, and was incubated at 37 °C for 15 min before each experiment. After the activated FRAP reagent was added to the solution of the tested compounds and the quercetin standard, the plate was incubated for 30 min. The absorbance was measured at 593 nm. The FRAP values are expressed as µM Ferrous equivalents calculated based on iron(II) sulfate heptahydrate calibration standard solutions. The experiments were run in duplicate and repeated three times.

It can be seen that compound **6c** was the most potent scavenger against the DPPH radical with the lowest IC_50_ value, followed by **6a**, **6d**, and **6b** at 200 µM concentrations These derivatives (**6a**, **6b**, **6c**, and **6d**) have two methoxy groups on the B ring. In a previous report, Kim B. T. et al. reported the effect of the substitution pattern of two hydroxyl groups on the B ring in the case of chalcones; the *ortho*- and *para*- substitution showed much stronger antioxidant potency than *meta*-substitution, due to the efficiency of the ortho- and para-dihydroxylated benzene ring system to delocalize electrons [27]. The steric hindrance is higher in the case of the three methoxy groups on the B ring, which perturbs planarity, causing the lower antioxidant potency of these derivatives (**6f** and **6e**) as the hydrogen abstraction is easier in planar geometrical configuration [28]. Moreover, in both cases, **6c** was the most potent compound, which bears the phenyl-*N*,*N*-dimethylamino group. These observations are in good correlation with Culhaoglu et al., who investigated the antioxidant activity of dialkylamino substituted 3-OH-flavon derivatives; the authors found that 4′-*N*,*N*-dimethylamino-3-OH-flavon showed significant scavenging of ABTS and DPPH radicals, and the potency was comparable to that of quercetin [20]. Furthermore, earlier, we observed that 4′-*N*,*N*-dimethylamino-flavon was also a good radical scavenger [18]. Thus, the presence of the phenyl-*N*,*N*-dimethylamino-group could have a crucial role in the radical scavenger activity of the molecules. Figure 3 shows the results of the FRAP assay of the investigated compounds expressed in µM ferrous equivalents.

**Figure 4:**
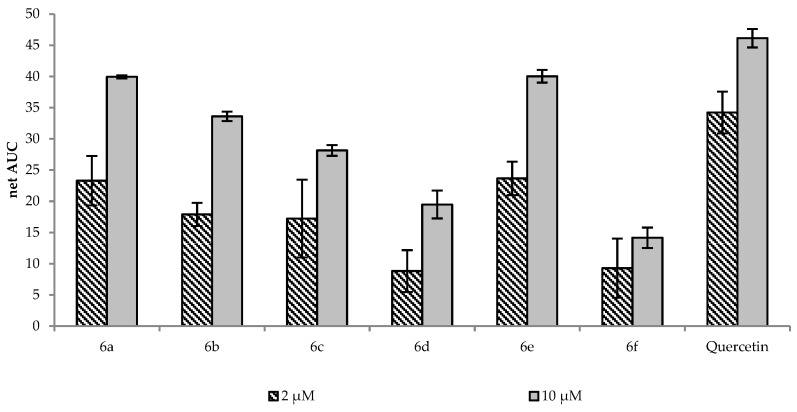
ORAC values of the compounds of interest expressed in the net area under the curve (AUC). To the mixture of tested compounds and fluorescein, the 2,2′-azobis(2-amidinopropane) dihydrochloride (AAPH) solution was added rapidly. The fluorescence was monitored at 485 nm excitation and 520 nm emission wavelength for 2 h in every 2 min. The assay was performed in duplicate and repeated three times. The netAUC was calculated by subtracting the blankAUC from sampleAUC.

The results show that compounds **6c**, **6a**, and **6e** had the highest FRAP values among the derivatives at all the tested concentrations, while compounds **6d**, **6f**, and **6b** exhibited significantly lower ferrous equivalents. It has to be mentioned that the FRAP value of each compound (**6a**–**f**) is significantly decreased compared to the standard quercetin. However, this result is not surprising, since it had been shown that methoxylated flavonoids are weaker antioxidants than the unmethylated ones [26,29]. In their study, Deng et al. found that quercetin had a 650-times higher FRAP value then the methylated form. In our experiments, we did not observe such a big difference, which could have originated from the presence of the free OH group at position 3 and the phenyl-*N*,*N*-dimethylamino group at ring A. Furthermore, since **6c**, **6e**, and **6a** showed a significantly higher FRAP value than **6b**, **6d**, and **6f** suggest that the presence of the phenyl-*N*,*N*-dimethylamino group is crucial, because the latter three compounds do not bear this group, but they also have the free OH group at position 3. Figure 4 depicts the results of the ORAC assay.

Similarly to the findings of Deng et al., the ORAC values did not change as much as the FRAP values [29]. The results show that compounds **6e**, **6a**, and **6b** had the highest ORAC value. Additionally, in the case of **6e** and **6a**, the results were comparable to quercetin at a 10 µM concentration. Surprisingly, compound **6c** exhibited only the fourth highest capacity for the absorption of the oxygen radical at each concentration.

### 2.3. Cytotoxic Effect

**Figure 5:**
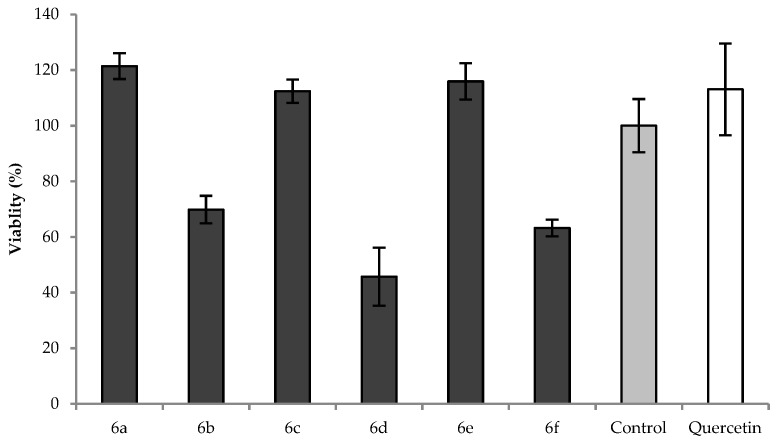
The effect of the tested compounds and the quercetin standard in 20 µM concentrations on the viability of H9c2 cells evaluated by the MTT assay. The cardiomyoblastoma cells were treated for 12 h and following the addition of the MTT solution, the plates were incubated for 4 h. Absorbance was measured at 540 and 630 nm. The measurements were carried out in triplicate and repeated two times.

Figure 5 presents the results of the MTT assay; the cells were treated with 20 µM flavonol solutions for 12 h. Compounds with the phenyl-*N*,*N*-dimethylamino functional group (**6a, 6c** and **6e**) did not exhibit cytotoxic activity; their cell viability percentage was comparable to the untreated control value and the quercetin standard.

The results are consistent with the findings of Luo W. et al., who found that 4-dimethylamine flavonoid derivatives are protective against oxidative stress-induced cell death in PC12 neurons [30]. Moreover, treatment with compounds **6b**, **6d**, and **6f** significantly decreased the viability of cells, which was the most dominant in the case of **6d**, which reduced the cell viability to 46% compared to the control. These results correlate well with the findings of other research groups who studied the effect of the number and position of methoxy groups on the flavonoid backbone on antioxidant and cytotoxic potency [24,29,31]. The presence of methoxy groups in these flavonols and other flavonoids could be the reason of their cytotoxicity depending on their number and/or positions; however, the exact structure-activity relationship is not completely understood.

### 2.4. Oxidative Transformation

**Scheme 2:**
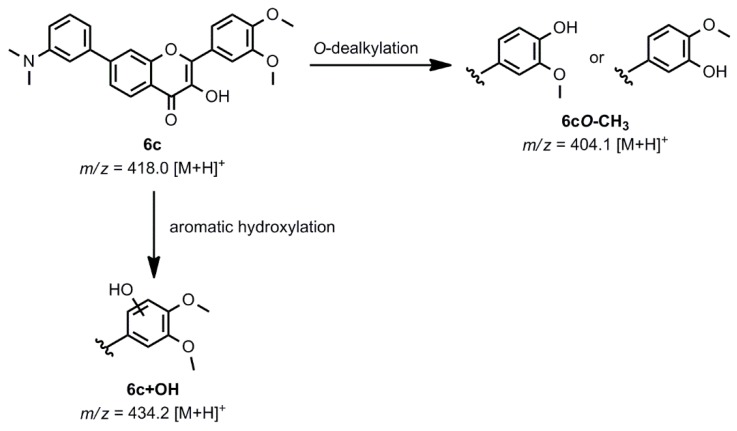
The oxidative transformation routes of compound **6c** based on the information obtained with the chemical Fenton system.

Based on the beneficial characteristics of **6c** demonstrated by the antioxidant and cytotoxicity assays, this compound was selected to determine the possible oxidative transformation pathways and identify its potential metabolites. The chemical Fenton reaction was used as a model of phase I biotransformation, since it is suitable for modeling phase I metabolic processes, such as *N*-dealkylation, *O*-dealkylation, *S*-oxidation, benzylic hydroxylation, and aromatic hydroxylation, as it has been shown previously [32,33]. Scheme 2 shows the oxidation routes and the potential metabolites of compound **6c** generated with the chemical Fenton system and detected with electrospray ionization mass spectrometry (ESI-MS, spectra not shown); an aromatic hydroxylation on the B ring resulted in the product **6c+OH** (*m/z* 434.2) and *O*-demethylation gave the most abundant **6cO-CH_3_** product (*m/z* 404.1) based on the peak intensity.

These results were also confirmed by LC-MS analysis. The place of the aromatic hydroxylation and *O*-dealkylation was deduced from the characteristic retro Diels-Alder rearrangement fragmentation pattern of the 3-hydroxyflavone backbone; however, due to the limitations of the detection method used, the exact positions are yet to be determined. The combination of electrochemical oxidation and the porphyrin system could yield additional oxidation products, such as secondary amine **6cN-CH_3_** after *N*-dealkylation, which would have the same *m/z* as **6cO-CH_3_**. The **6cN-CH_3_** could undergo another *N*-dealkylation, which results in the **6cN-2CH_3_** primary amine.

## 3. Materials and Methods

### 3.1. Chemistry

#### 3.1.1. General Information

Thin-layer chromatography with aluminium backed TLC plates of silica gel 60 F254 (0.2 mm, Merck, Darmstadt, Germany) under UV light was used to monitor the reactions. Column chromatography was performed on silica gel (60, 70–230 mesh, Merck, Darmstadt, Germany). Melting points were determined by Büchi B-540 apparatus (Büchi Labortechnik AG, Flawil, Switzerland). The purity of the compounds was evaluated using an LTQ XL linear ion trap mass spectrometer (Thermo Fisher Scientific, Waltham, MA, USA) with positive ESI. ^1^H-NMR and ^13^C-NMR spectra were acquired on a Bruker AM 360 (360.13 MHz for ^1^H, 90.03 MHz for ^13^C) spectrometer (Bruker, Billerica, MA, USA). Chemical shifts (δ) are given from internal CHCl_3_ (δ = 7.26 ppm) or TMS (δ = 0.00 ppm) signals for ^1^H-NMR and CHCl_3_ (δ = 77.00 ppm) or DMSO (δ = 39.52 ppm) for ^13^C-NMR and reported in parts per million (ppm, d). Coupling constants (J) were measured and reported in hertz (Hz). Spectra can be found in Supplementary Materials. Elemental analyses (C, H, N) were performed using the Elementar Vario MicroCube instrument (Elementar Analysensysteme GmbH, Langenselbold, Germany). Infrared spectra were obtained in KBr discs using a JASCO FT-IR 4100A Fourier-transform infrared spectrometer (Jasco Inc, Easton, MD, USA).

#### 3.1.2. General Procedure for the Synthesis of **3a**–**c**

To the stirred solution of the acetophenone (**1a**,**b**, 1.29 g, 6 mmol) in MeOH (10 mL) 50% aq. NaOH (1.26 mL, 24 mmol) was added. Benzaldehyde (**2a**,**b**, 6.3 mmol) suspension in MeOH (5 mL) was added to the solution of the acetophenone and the mixture was stirred for 1 h, and then it was allowed to stand at room temperature for one day. HCl solution (10%, w/v) was added to reach pH 1; the precipitate was filtered off and washed with water (3 × 30 mL) to give **3a**–**c** (88–97%).

*(E)-1-(5-Bromo-2-hydroxyphenyl)-3-(3,4-dimethoxyphenyl)prop-2-en-1-one* (**3a**). ^1^H NMR (300 MHz, 298 K, CDCl_3_): δ (ppm) = 3.95–3.98 (m, 6H, OMe), 6.89–6.93 (m, 2H, 5-H, 3′-H), 7.17 (s, 1H, 2-H), 7.27–7.29 (d, J = 7.35 Hz, 1H, 6-H), 7.37-7.41 (d, J = 15.25 Hz, 1H, α-H), 7.53–7.56 (dd, 1H, 4′-H), 7.87–7.92 (d, J = 15.34 Hz, 1H, β-H), 8.00–8.00 (d, J = 1.62 Hz, 6′-H), 12.89 (s, 1H, OH).

*(E)-1-(4-Bromo-2-hydroxyphenyl)-3-(3,4-dimethoxyphenyl)prop-2-en-1-one* (**3b**). ^1^H NMR (360 MHz, 298 K, CDCl_3_): δ (ppm) = 3.94–3.96 (m, 6H, OMe), 6.89–6.91 (d, J = 8.36 Hz, 1H, 5-H), 7.03–7.06 (dd, 1H, 6-H), 7.14–7.19 (m, 2H, 2-H, 3′-H), 7.23–7.26 (dd, 1H, 5′-H), 7.38–7.42 (d, J = 15.51 Hz, 1H, α-H), 7.73–7.76 (d, J = 8.36 Hz, 1H, 6′-H), 7.85-7.89 (d, J = 15.63 Hz, 1H, β-H), 13.09 (s, 1H, OH).

*(E)-1-(4-Bromo-2-hydroxyphenyl)-3-(3,4,5-trimethoxyphenyl)prop-2-en-1-one* (**3c**). ^1^H NMR (400 MHz, 298 K, CDCl_3_): δ (ppm) = 3.92–3.93 (m, 9H, OMe), 6.87 (s, 2H, 2-H, 6-H), 7.05–7.07 (d, J = 7.70 Hz, 1H, 5′-H), 7.20 (s, 1H, 3′-H), 7.41–7.45 (d, J = 15.40 Hz, 1H, α-H), 7.74–7.77 (d, J = 7.70 Hz, 1H, 6′-H), 7.82–7.86 (d, J = 15.40 Hz, 1H, β-H), 12.99 (s, 1H, OH).

#### 3.1.3. General Procedure for the Synthesis of **4a**–**c**

To the water-bath cooled suspension of chalcone (**3a**–**c**, 2.7 mmol) in EtOH (15 mL), 8% aq. NaOH (3.9 mL, 8.41 mmol) was added to result in a solution. To the mixture, 30% H_2_O_2_ (3.9 mL, 38.2 mmol) was added dropwise, and then it was stirred at room temperature for 2 h. The reaction mixture was poured into ice-water mixture (250 mL) and 10% HCl solution was added to reach pH 1. The precipitate was allowed to sedimentate for one day, then filtered off and washed with cc. NaHCO_3_ solution (2 × 50 mL) and water (4 × 50 mL) to give **4a**–**c** (57–76%).

*6-Bromo-2-(3,4-dimethoxyphenyl)-3-hydroxy-4H-chromen-4-one* (**4a**). ^1^H NMR (360 MHz, 298 K, DMSO-D_6_): δ (ppm) = 3.85 (s, 6H, OMe), 7.14–7.16 (d, J = 7.85 Hz, 1H, 5′-H), 7.80–7.82 (m, 3H, 2′-H, 6′-H), 7.87–7.90 (d, J = 8.16 Hz, 1H, 8-H), 7.93–7.96 (dd, 7-H), 8.16–8.16 (d, J = 1.92 Hz, 1H, 5-H), 9.72 (s, 1H, OH).

*7-Bromo-2-(3,4-dimethoxyphenyl)-3-hydroxy-4H-chromen-4-one* (**4b**).^1^H NMR (400 MHz, 298 K, DMSO-D_6_): δ (ppm) = 3.85–3.86 (m, 6H, OMe), 7.14–7.16 (d, J = 8.02 Hz, 1H, 5′-H), 7.61–7.64 (dd, 1H, 6′-H) 7.81–7.81 (d, J = 1.67 Hz, 1H, 2′-H), 7.90–7.93 (dd, 1H, 6-H), 8.00–8.02 (d, J = 8.55 Hz, 5-H), 8.20–8.20 (d, J = 1.44 Hz, 1H, 8-H), 9.63 (s, 1H, OH).

*7-Bromo-3-hydroxy-2-(3,4,5-trimethoxyphenyl)-4H-chromen-4-one* (**4c**). ^1^H NMR (400 MHz, 298 K, DMSO-D_6_): δ (ppm) = 3.75 (s, 3H, 4′-MeO), 3.86 (s, 6H, 3′-MeO, 5′-MeO), 7.56 (s, 2H, 2′-H, 6′-H), 7.59–7.62 (d, J = 8.46 Hz, 1H, 6-H), 7.98–8.00 (d, J = 8.46 Hz, 5-H), 8.21 (s, 1H, 8-H), 9.74 (s, 1H, OH).

#### 3.1.4. General Procedure for the Synthesis of **6a**–**f**

Into a pressure tube, under argon, the mixture of 3-hydroxyflavone (**4a**–**c**, 0.265 mmol), KF (46.3 mg, 0.795 mmol), Pd(OAc)_2_ (3 mg, 0.0133 mmol) and XPhos (12.6 mg, 0.0265 mmol), and boronic acid (**5a**,**b**, 0.53 mmol) in toluene/t-BuOH (6:1, 3.5 mL) were added. The mixture was stirred and heated in a 100 °C oil bath for 4 h. The solvent was removed under reduced pressure, and the residue was purified by absorptive filtration using toluene/EtOAc (2:1) as the eluent. The crude product was washed with *i*-Pr_2_O and filtered to give pure products **6a**–**f** (63–74%).

*2-(3,4-Dimethoxyphenyl)-6-[3-(dimethylamino)phenyl]-3-hydroxy-4H-chromen-4-one* (**6a**). Yellow solid; yield 69.1 mg (63%). Mp. 187.7–188.8 °C. Rf: 0.35 (toluene/EtOAc, 2:1). ^1^H NMR (360 MHz, 298 K, CDCl_3_): δ (ppm) = 3.02 (s, 6H, N(Me)_2_), 3.95–3.99 (m, 6H, OMe), 6.75–6.77 (d, J = 7.35 Hz, 1H, 6″-H), 6.97–6.99 (m, 3H, 8-H, 5′-H, 2″-H), 7.13–7.17 (m, 1H, 2′-H), 7.30–7.34 (m, 1H, 5″-H), 7.60 (s, 1H, OH), 7.85–7.90 (m, 3H, 7-H, 6′-H, 4″-H), 8.42 (s, 1H, 5-H). ^13^C NMR (91 MHz, 298 K, CDCl_3_): δ (ppm) = 40.8 (C-N(Me)_2_), 56.0, 56.1 (C-3′-MeO, 4′-MeO), 110.8 (C-2′), 111.1 (C-5′), 111.3 (C-2″), 112.2 (C-4″), 115.7 (C-6″), 118.5 (C-8), 120.8 (C-1′), 121.6 (C-6′), 123.1 (C-5), 123.8 (C-4a), 129.7 (C-5″), 132.8 (C-7), 137.9 (C-3), 138.7 (C-6), 140.3 (C-1″), 145.2 (C-4′), 148.9 (C-3′), 150.8(C-3″), 151.1 (C-2), 154.6 (C-8a),173.3 (C-4). LC-MS: *m/z* = 418.42 [M + H]^+^. IR (KBr, cm^−1^): ν = 3292, 2934, 2839, 1605, 1561, 1516, 1489, 1463.71, 1430, 1381, 1338, 1271, 1247, 1216, 1195, 1149, 1118, 1042, 1024, 991, 964, 933, 901, 869, 814, 770, 724, 694, 665, 634. Anal. Calcd for C_25_H_23_NO_5_: C, 71.93; H, 5.55; Found: C 71.79; H 5.52.

*2-(3,4-Dimethoxyphenyl)-3-hydroxy-6-[4-(methoxymethyl)phenyl]-4H-chromen-4-one* (**6b**). Yellow solid; yield 80.3 mg (72%). Mp. 192.8–194.3 °C. Rf: 0.30 (toluene/EtOAc, 2:1). ^1^H NMR (360 MHz, 298 K, CDCl_3_): δ (ppm) = 3.43 (s, 3H, Bn-MeO), 3.96–4.00 (m, 6H, 3′-MeO, 4′-MeO), 4.52 (s, 2H, Bn-H_2_), 7.00–7.02 (d, J = 7.37 Hz, 1H, 5′-H), 7.08 (s, 1H, 2′-H), 7.43–7.45 (d, J = 7.55 Hz, 2H, 2″-H, 6″-H), 7.62–7.69 (m, 3H, OH, 3″-H, 5″-H), 7.86–7.93 (m, 3H, 7-H, 8-H, 6″-H), 8.43 (s, 1H. 5-H). ^13^C NMR (91 MHz, 298 K, CDCl_3_): δ (ppm) = 56.1, 56.1 (C-3′-MeO, 4′-MeO), 58.4 (C-Bn-MeO), 74.4 (C-Bn-CH_2_), 110.9 (C-2′), 111.1 (C-5′), 118.8 (C-8), 120.9 (C-1′), 121.6 (C-6′), 123.1 (C-5), 123.8 (C-4a), 127.3 (C-2″, C-6″), 128.4 (C-3″, C-5″), 132.4 (C-7), 137.4 (C-4″),138.0 (C-3), 138.1 (C-6), 138.7 (C-1″), 145.2 (C-4′), 149.0 (C-3′), 150.9 (C-2), 154.7 (C-8a), 173.2 (C-4). LC-MS: *m/z* = 419.33 [M + H]^+^. IR (KBr, cm^−1^): ν = 3265, 3030, 2993, 2939, 2833, 2737, 2603, 2039, 1922, 1843, 1801, 1714, 1601, 1561, 1516, 1483, 1459, 1411, 1388, 1336, 1298, 1269, 1218, 1197, 1174, 1148, 1112, 1025, 967, 944, 932, 904, 875, 857, 815, 806, 784, 770, 727, 699, 661, 629. Anal. Calcd for C_25_H_22_O_6_: C, 71.76; H, 5.30; Found: C 71.95; H 5.29.

*2-(3,4-Dimethoxyphenyl)-7-[3-(dimethylamino)phenyl]-3-hydroxy-4H-chromen-4-one* (**6c**). Pale-brown solid; yield 79.0 mg (71%). Mp. 191.6moved outside the brackets192.7 °C. Rf: 0.39 (toluene/EtOAc, 2:1). ^1^H NMR (400 MHz, 298 K, CDCl_3_): δ (ppm) = 3.04 (s, 6H, N(Me)_2_), 3.96–3.99 (m, 6H, OMe), 6.79–6.81 (d, J = 7.84 Hz, 1H, 6″-H), 6.97–7.02 (m, 3H, 8-H, 5′-H, 2″-H), 7.10 (s, 1H, 2′-H), 7.33–7.37 (m, 1H, 5″-H), 7.63–7.65 (d, J = 7.84 Hz, 1H, 6-H), 7.75 (s, 1H, OH), 7.87–7.91 (m, 2H, 6′-H, 4″-H), 8.24–8.26 (d, J = 7.83 Hz, 1H, 5-H). ^13^C NMR (101 MHz, 298 K, CDCl_3_): δ (ppm) = 40.8 (C-N(Me)_2_), 56.1, 56.2 (C-3′-MeO, 4′-MeO), 110.9 (C-2′), 111.0 (C-5′), 111.4 (C-2″), 112.9 (C-4″), 115.9 (C-6″), 116.2 (C-8), 119.4 (C-1′), 121.5 (C-6′), 123.9 (C-4a), 124.1 (C-6), 125.7 (C-5), 129.8 (C-5″), 138.0 (C-3), 140.3 (C-1″), 145.3 (C-4′), 147.8 (C-7), 149.0 (C-3′), 150.8 (C-3″), 151.1 (C-2), 155.6 (C-8a), 173.0 (C-4). LC-MS: *m/z* = 418.42 [M + H]^+^. IR (KBr, cm^−1^): ν = 3228, 3028, 2992, 2919, 2847, 2800, 2592, 2034, 1940, 1885, 1713, 1617, 1576, 1555, 1514, 1489, 1453, 1428, 1397, 1346, 1265, 1236, 1213, 1171, 1147, 1111, 1039, 1024, 994, 969, 921, 883, 861, 847, 835, 821, 771, 717, 699, 648, 639. Anal. Calcd for C_25_H_23_NO_5_: C, 71.93; H, 5.55; Found: C 71.97; H 5.52.

*2-(3,4-Dimethoxyphenyl)-3-hydroxy-7-[4-(methoxymethyl)phenyl]-4H-chromen-4-one* (**6d**). Yellow solid; yield 79.3 mg (72%). Mp. 212.1–214.2 °C. Rf: 0.31 (toluene/EtOAc, 2:1). ^1^H NMR (400 MHz, 298 K, CDCl_3_): δ (ppm) = 3.44 (s, 3H, Bn-MeO), 3.96–4.00 (m, 6H, 3′-MeO, 4′-MeO), 4.53 (s, 2H, Bn-H_2_), 6.99–7.01 (d, J = 7.46 Hz, 1H, 5′-H), 7.10 (s, 1H, 2′-H), 7.46–7.48 (m, 2H, 2″-H, 6″-H), 7.62–7.69 (m, 3H, 6-H, 3″-H, 5″-H), 7.75 (s, 1H, OH), 7.85–7.91 (m, 2H, 8-H, 6′-H), 8.25–8.27 (d, J = 7.46 Hz, 1H, 5-H). ^13^C NMR (101 MHz, 298 K, CDCl_3_): δ (ppm) = 56.1, 56.1 (C-3′-MeO, 4′-MeO), 58.4 (C-Bn-MeO), 74.3 (C-Bn-CH_2_), 110.8 (C-2′), 111.0 (C-5′), 116.0 (C-8), 119.5 (C-1′), 121.6 (C-6′), 123.7 (C-6), 123.8 (C-4a), 125.9 (C-5), 127.5 (C-2″, C-6″), 128.4 (C-3″, C-5″), 138.0 (C-3), 138.5 (C-4″), 139.1 (C-1″), 145.3 (C-4′), 146.3 (C-7), 149.0 (C-3′), 150.8 (C-2), 155.6 (C-8a), 172.9 (C-4). LC-MS: *m/z* = 419.42 [M + H]^+^. IR (KBr, cm^−1^): ν = 3234, 3022, 2977, 2920, 2839, 2597, 2030, 1923, 1848, 1797, 1714, 1611, 1574, 1555, 1515, 1488, 1467, 1450, 1417, 1397, 1354, 1333, 1271, 1243, 1212, 1173, 1147, 1103, 1039, 1020, 914, 858, 843, 818, 807, 774, 706, 635. Anal. Calcd for C_25_H_22_O_6_: C, 71.76; H, 5.30; Found: C 71.70; H 5.32.

*7-[3-(Dimethylamino)phenyl]-3-hydroxy-2-(3,4,5-trimethoxyphenyl)-4H-chromen-4-one* (**6e**). Pale-brown solid; yield 88.2 mg (74%). Mp. 179.5–181.8 °C. Rf: 0.47 (toluene/EtOAc, 2:1). ^1^H NMR (360 MHz, 298 K, CDCl_3_): δ (ppm) = 3.04 (s, 6H, N(Me)_2_), 3.95 (s, 3H, 4′-MeO), 3.97 (s, 6H, 3′-MeO, 5′-MeO), 6.80–6.82 (d, J = 7.17 Hz, 1H, 6″-H), 6.97–7.03 (m, 2H, 8-H, 2″-H), 7.15–7.17 (d, J = 7.17 Hz, 1H, 4″-H), 7.34–7.37 (m, 1H, 5″-H), 7.56 (s, 2H, 2′-H, 6′-H), 7.64–7.66 (d, J = 7.96 Hz, 1H, 6-H), 7.76 (s, 1H, OH), 8.25–8.27 (d, J = 8.22 Hz, 1H, 5-H). ^13^C NMR (91 MHz, 298 K, CDCl_3_): δ (ppm) = 40.8 (C-N(Me)_2_), 56.4 (C-3′-MeO, 5′-MeO), 61.1 (C-4′), 105.6 (C-2′ C-6′), 111.5 (C-2″), 113.0 (C-4″), 115.9 (C-6″), 116.3 (C-8), 119.3 (C-1′), 126.4 (C-4a), 124.3 (C-6), 125.7 (C-5), 129.9 (C-5″), 138.4 (C-3), 140.2 (C-1″), 144.8 (C-4′), 148.1 (C-7), 151.1 (C-2), 151.2 (C-3″), 153.3 (C-3′, C-5′), 155.7 (C-8a), 173.2 (C-4). LC-MS: *m/z* = 448.33 [M + H]^+^. IR (KBr, cm^−1^): ν = 3247, 3079, 2999, 2968, 2938, 2838, 1603, 1579, 1556, 1504, 1453, 1427, 1393, 1353, 1283, 1263, 1242, 1209, 1172, 1127, 1057, 1027, 1011, 994, 928, 841, 831, 821, 773, 734, 714, 697, 652. Anal. Calcd for C_26_H_25_NO_6_: C, 69.79; H, 5.63; Found: C 69.86; H 5.59.

*3-Hydroxy-7-[4-(methoxymethyl)phenyl]-2-(3,4,5-trimethoxyphenyl)-4H-chromen-4-one* (**6f**). Brown solid; yield 82.0 mg (69%). Mp. 208.1–209.2 °C. Rf: 0.48 (toluene/EtOAc, 2:1). ^1^H NMR (400 MHz, 298 K, CDCl_3_): δ (ppm) = 3.45 (s, 3H, Bn-MeO), 3.95 (s, 3H, 4′-MeO), 3.98 (s, 6H, 3′-MeO, 5′-MeO), 4.54 (s, 2H, Bn-H_2_), 7.15 (s, 1H, 8-H), 7.48 (s, 2H, 2′-H, 6′-H), 7.56 (s, 2H, 2″-H, 6″-H), 7.63–7.69 (m, 3H, 6-H, 3″-H, 5″-H), 7.77 (s, 1H, OH), 8.26–8.28 (d, J = 7.47 Hz, 1H, 5-H). ^13^C NMR (101 MHz, 298 K, CDCl_3_): δ (ppm) = 56.4 (C-3′-MeO, 5′-MeO), 58.4 (C-Bn-MeO), 61.1 (C-4′), 74.3 (C-Bn-CH_2_), 105.5 (C-2′), 116.1 (C-8), 119.4 (C-1′), 123.9 (C-6), 126.4 (C-4a), 126.0 (C-5), 127.5 (C-2″, C-6″), 128.4 (C-3″, C-5″), 138.4 (C-3), 138.5 (C-4″), 139.2 (C-1″), 140.1 (C-2), 144.8 (C-4′), 146.6 (C-7), 153.3 (C-3′, C-5′), 155.7 (C-8a), 173.1 (C-4). LC-MS: *m/z* = 449.33 [M + H]^+^. IR (KBr, cm^−1^): ν = 3278, 3010, 2983, 2969, 2939, 2899, 2837, 2154, 2064, 2010, 1928, 1602, 1583, 1574, 1551, 1508, 1491, 1451, 1409, 1394, 1379, 1338, 1296, 1244, 1211, 1194, 1172, 1130, 1114, 1056, 1026, 1011, 970, 936, 930, 913, 887, 844, 824, 779, 765, 712, 671, 624. Anal. Calcd for C_26_H_24_O_7_: C, 69.63; H, 5.39; Found: C 69.57; H 5.43.

### 3.2. Antioxidant Activity and Cytotoxicity

#### 3.2.1. Chemicals

Water (ultra-pure) was prepared with the SolPure 78 water purification system from POL-LAB (Bielsko-Biała, Poland). Ethylenediaminetetraacetic acid disodium salt dihydrate (EDTA-Na_2_), L(+)-ascorbic acid, ethanol (96%), acetic acid, and formic acid were obtained from Scharlab Magyarország Kft. (Debrecen, Hungary). DMSO, methanol, hydrogen peroxide solution, 2,2-diphenyl-1-picrylhydrazyl (DPPH), glacial acetic acid, sodium acetate, HCl, 2,4,6-tri(2-pyridyl)-s-triazine (TPTZ), FeCl_3_, iron(II) sulfate heptahydrate, fluorescein, 2,2′-azobis (2-amidinopropane) dihydrochloride (AAPH), 3-(4,5-dimethylthiazol-2-yl)-2,5-diphenyltetrazolium bromide (MTT), potassium hydrogen phosphate, monopotassium phosphate, and K_2_S_2_O_8_ were obtained from Sigma-Aldrich Kft. (Budapest, Hungary). Acetonitrile (ACN) was ordered from Merck (Darmstadt, Germany). 2,2′-Azino-bis(3-ethylbenzothiazoline-6-sulphonic acid) diammonium salt (ABTS salt) was purchased from Fluorochem Ltd. (Hadfield, United Kingdom).

#### 3.2.2. ABTS Assay

To measure the ABTS radical scavenging activity, the work of Sugahara et al. and Re et al. was followed closely, applying few alterations [34,35]. A mixture containing 7 mM ABTS and 2.45 mM K_2_S_2_O_8_ in water was kept in the dark for 16 h to form ABTS^•+^. The working solution was prepared by adding 150 µL of ABTS^•+^ solution to 2.9 mL MeOH. Aliquots of 180 µL of this working solution were pipetted into wells containing 20 µL of test samples in DMSO with different concentrations (10, 20, 50, 100, and 200 µM). Absorbance was measured at 737 nm after 10 s mixing using a Multiskan GO microplate spectrophotometer (Thermo Fisher Scientific, Waltham, MA, USA) at 0, 1, 2, 3, 4, 5, 10, 15, 30, 45, 60, 75, 90, 105, and 120 min. Quercetin was used as the standard. The IC_50_ values were calculated based on the inhibition percentage measured at 120 min. The experiments were carried out in duplicate and repeated three times.

#### 3.2.3. DPPH Assay

The method used for the DPPH radical scavenging assay was adopted from Clarke et al., with modifications [36]. A total of 180 µL freshly made 0.2 mM DPPH solution in methanol was added to 20 µL of the investigated compounds in various concentrations (10, 20, 50, 100, and 200 µM) in DMSO. After a gentle, 10-s mixing period, the plate was kept in the dark for 90 min. and the absorbance was read at 515 nm using a Multiskan GO microplate spectrophotometer (Thermo Fisher Scientific, Waltham, MA, USA) at 0, 1, 2, 3, 4, 5, 10, 15, 30, 45, 60, 75, and 90 min. Blanks (20 µL DMSO with 180 µL MeOH) and quercetin standards in the same concentration as the tested compounds were measured simultaneously. The IC_50_ values were calculated based on the inhibition percentage measured at 90 min. The measurements were performed in duplicate and repeated three times.

#### 3.2.4. FRAP Assay

The ferric reducing antioxidant power of the compounds was tested with a modified method of Benzie and Strain [37]. The FRAP reagent consisted of 10 mL acetate buffer (300 mM, pH 3.6), 1 mL TPTZ solution (10 mM) in 40 mM HCl, and 1 mL FeCl_3_ (10 mM). The mixture was incubated at 37 °C for 15 min and used on the same day. The reaction mixture was prepared by 20 µL of the investigated compound (10, 20, 50, 100, and 200 µM) and 180 µL activated FRAP reagent in a 96-well plate. Following a 30 min incubation period, the absorbance was measured at 593 nm by a Multiskan GO microplate spectrophotometer (Thermo Fisher Scientific, Waltham, MA, USA). Iron (II) sulfate heptahydrate was used as the standard in different concentrations (0.5, 1, 5, 10, 50, 100, and 500 µM). The FRAP values are expressed as µM ferrous equivalents. The experiments were run in duplicate and repeated three times.

#### 3.2.5. ORAC Assay

The oxygen radical absorbance capacity was evaluated using the ORAC assay. The test was carried out in a black 96-well plate with a black bottom. The stock solutions of 50 nM fluorescein and 180 mM AAPH in phosphate buffer (75 mM, pH 7.00) were prepared freshly and used on the same day. To each well, 20 µL of compound in ACN (2 and 10 µM) was added to 160 µL fluorescein. In the case of blank samples, 20 µL of ACN was added. The plate was incubated for 15 min at 37 °C, and then the 20 µL AAPH solution was rapidly added to each well to initiate the reaction. The fluorescence was monitored at 485 nm excitation and 520 nm emission wavelength for 2 h every 2 min by the FLUOstar OPTIMA (BMG Labtech, Ortenberg, Germany) plate reader. The assay was performed in duplicate and repeated three times. The area under the curve (AUC) was calculated as follows:

AUC = 0.5 + (A_1_/A_0_) + (A_2_/A_0_) + (A_3_/A_0_) + ⋯ + (A_n_/A_0_), (1)

where A_0_ is the fluorescence reading at 0 min and A_n_ is the fluorescence reading at n min. The reported ORAC values were calculated by subtracting the blank AUC from the sample AUC and expressed as AUC_net_.

AUC_net_ = AUC_sample_ − AUC_blank_ (2)

#### 3.2.6. MTT Assay

The MTT assay was used to determine the effect of the investigated compounds on the cell viability. H9c2 cells (ATCC, CRL-1446, LGC Standards GmbH, Wesel, Germany) were seeded into 96-well plates at a density of 6000 cells/well in 200 µL media (DMEM, high glucose, pyruvate from Thermo Fischer Scientific with 10% FBS and supplemented with penicillin and streptomycin) and cultured overnight. The flavonols were added to the cells in 20 µM concentration. After 12 h, 20 μL of MTT solution (5 mg/μL in PBS) was added to each well, and the plates were incubated for 4 h. Afterwards, the media was carefully removed and 100 μL DMSO was added to each well to dissolve the formazan crystals. Absorbance was measured at 540 and 630 nm using a Synergy HT plate reader (BioTek, Winooski, VT, USA). The tests were carried out in triplicate and repeated two times.

### 3.3. Oxidative Transformation

#### Chemical Fenton Reaction

The Fenton reaction of the compounds was carried out based on a method reported previously by Csepanyi et al. [18]. The reaction mixture consisted of 400 μL 2.5 mM of the investigated compound in ACN, 50 μL 20 mM FeCl_3_, 50 μL 20 mM EDTA-Na_2_, and 500 μL 10 mM ascorbic acid in ACN/H_2_O (50:50) and 2 μL 30% hydrogen peroxide. The reaction mixture was stirred at room temperature at 700 rpm. Samples were drawn at 2, 24, 72, and 144 h and were diluted with ACN/H_2_O (50:50) and analyzed immediately by an API 2000 Triple Quadrupole mass spectrometer (Applied Biosystems, Waltham, MA, USA) equipped with a syringe pump with the following parameters: 20 μL/min flow rate, 10 PSI curtain gas, 20 V declustering potential, 4000 V ion spray voltage, 400 V focusing potential, and 200 °C ion source temperature. The positive ion electrospray mass spectra were recorded in the range of *m*/*z* 100–500 using Analyst 1.5.1. Software (AB SCIEX, Concord, ON, Canada). In additional experiments, the reaction mixture was separated and analyzed with an LTQ XL linear ion trap mass spectrometer coupled with the Accela LC system (Thermo Fisher Scientific, Waltham, MA, USA). The HPLC separation was performed using a Kinetex XB-C18 2.6 μm column, 0.1% formic acid in water, and ACN with 0.1% formic acid with gradient elution, and the flow rate was set to 300 μL/min. The method parameters for mass spectrometry were the followings: 35 a.u. sheath gas flow rate, 5000 V spray voltage, 300 °C capillary temperature, 31 V capillary voltage, 150 V tube lens voltage, and 34 V skimmer voltage.

## 4. Conclusions

In conclusion, six new flavonol derivatives were designed and synthetized, and their biological activity was evaluated. The results of four antioxidant assays showed that three compounds (**6a**, **6c**, and **6e**) were potent antioxidants. Each of these molecules bear with a phenyl-*N*,*N*-dimethylamino group on ring A. Furthermore, compounds **6a**, **6c**, and **6e** did not show any cytotoxic activity on H9c2 cells during the MTT assay, when 20 µM solutions were tested. According to the experimental data, the position of the phenyl-*N*,*N*-dimethylamino group did not significantly affect the antioxidant potential. However, because compound **6c** showed improved antioxidant activity among compounds with a phenyl-*N*,*N*-dimethylamino substituent, this molecule was selected for further studies, in which the oxidative biotransformation of the molecule was modeled by a biomimetic system; the chemical Fenton reaction. As a result of these experiments, two potential metabolites were detected, which were the products of *O*-demethylation and aromatic hydroxylation of compound **6c**. These results identify compound **6c** as a potential subject of additional investigations and a prototype of promising flavonol-type antioxidants in further developments. Furthermore, to the authors’ best knowledge, this is the first time that the dimethylamino group has been proven to be involved in the antioxidant activity in flavonols.

## Figures and Tables

**Table 1:**
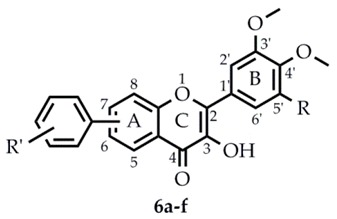
The structures of the investigated flavonol derivatives.

ID	R	R’	Position of R‘-Ph
**6a**	H	3-N(CH_3_)_2_	6
**6b**	H	4-CH_2_(O)CH_3_	6
**6c**	H	3-N(CH_3_)_2_	7
**6d**	H	4-CH_2_(O)CH_3_	7
**6e**	OMe	3-N(CH_3_)_2_	7
**6f**	OMe	4-CH_2_(O)CH_3_	7

## Supplementary Materials

The supplementary materials are available online.
