# Supplementary material for: Synthesis, In Vitro Biological Evaluation, and Oxidative Transformation of New Flavonol Derivatives: The Possible Role of the Phenyl-N,N-Dimethylamino Group
Source: Molecules. 2018 Nov 30;23(12):3161. doi: 10.3390/molecules23123161 (PMC6320925; doi:10.3390/molecules23123161)

(*E*)-1-(5-bromo-2-hydroxyphenyl)-3-(3,4-dimethoxyphenyl)prop-2-en-1-one (**3a**)

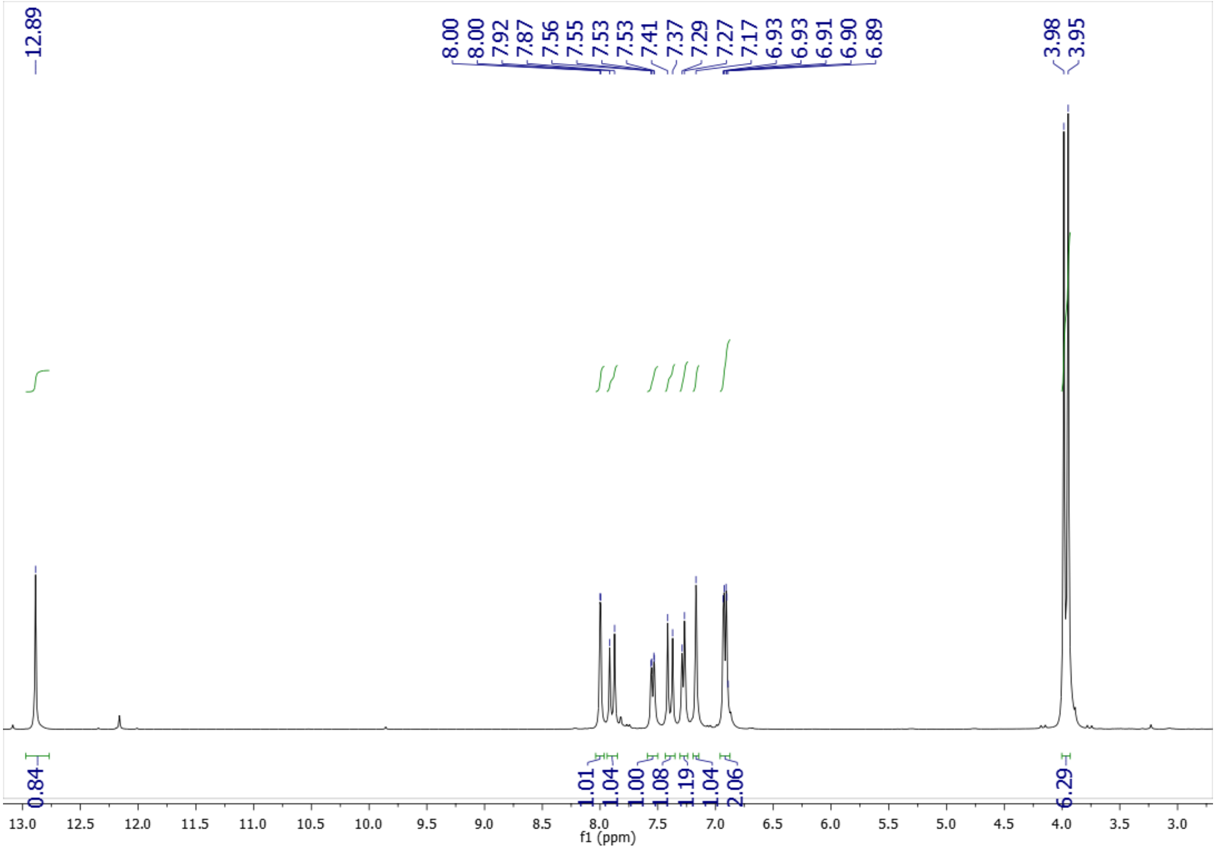

(*E*)-1-(4-bromo-2-hydroxyphenyl)-3-(3,4-dimethoxyphenyl)prop-2-en-1-one (**3b**)

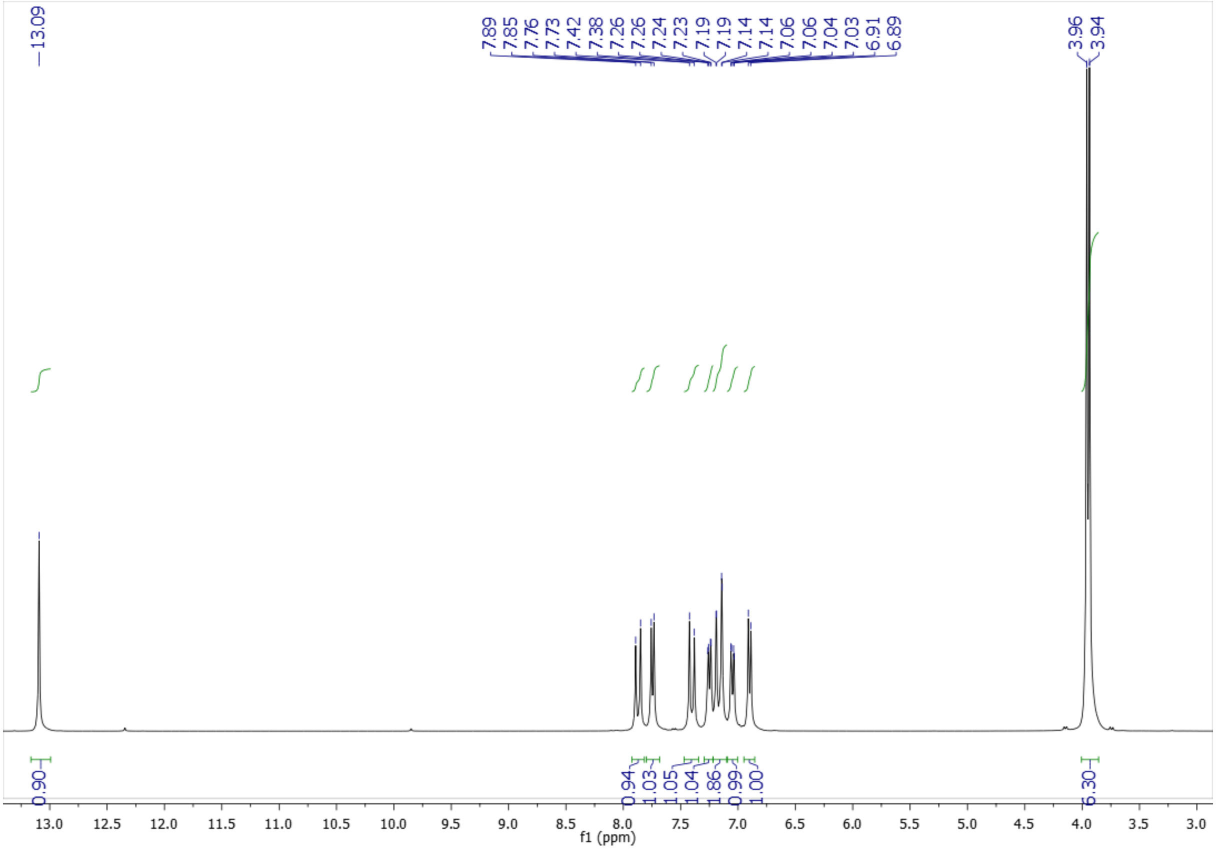

(*E*)-1-(4-bromo-2-hydroxyphenyl)-3-(3,4,5-trimethoxyphenyl)prop-2-en-1-one (**3c**)

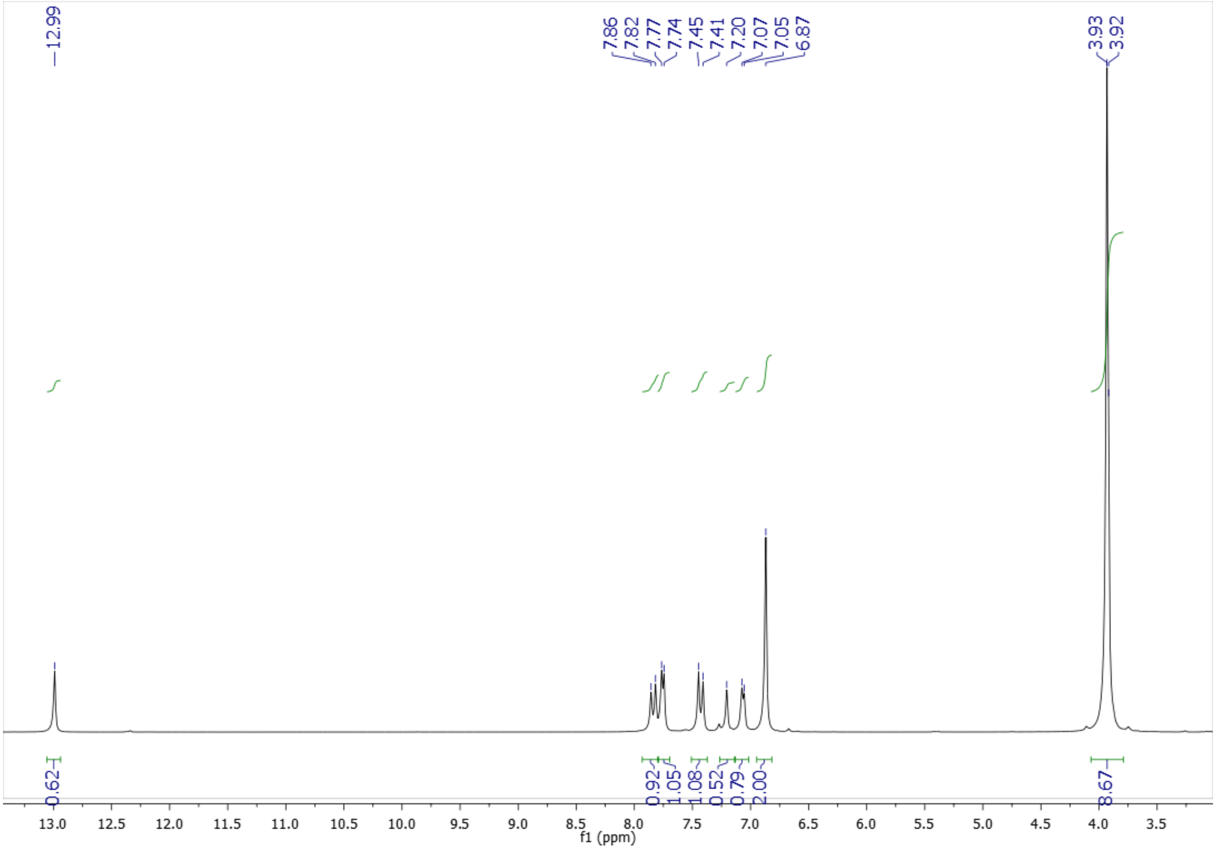

6-Bromo-2-(3,4-dimethoxyphenyl)-3-hydroxy-4H-chromen-4-one (**4a**)

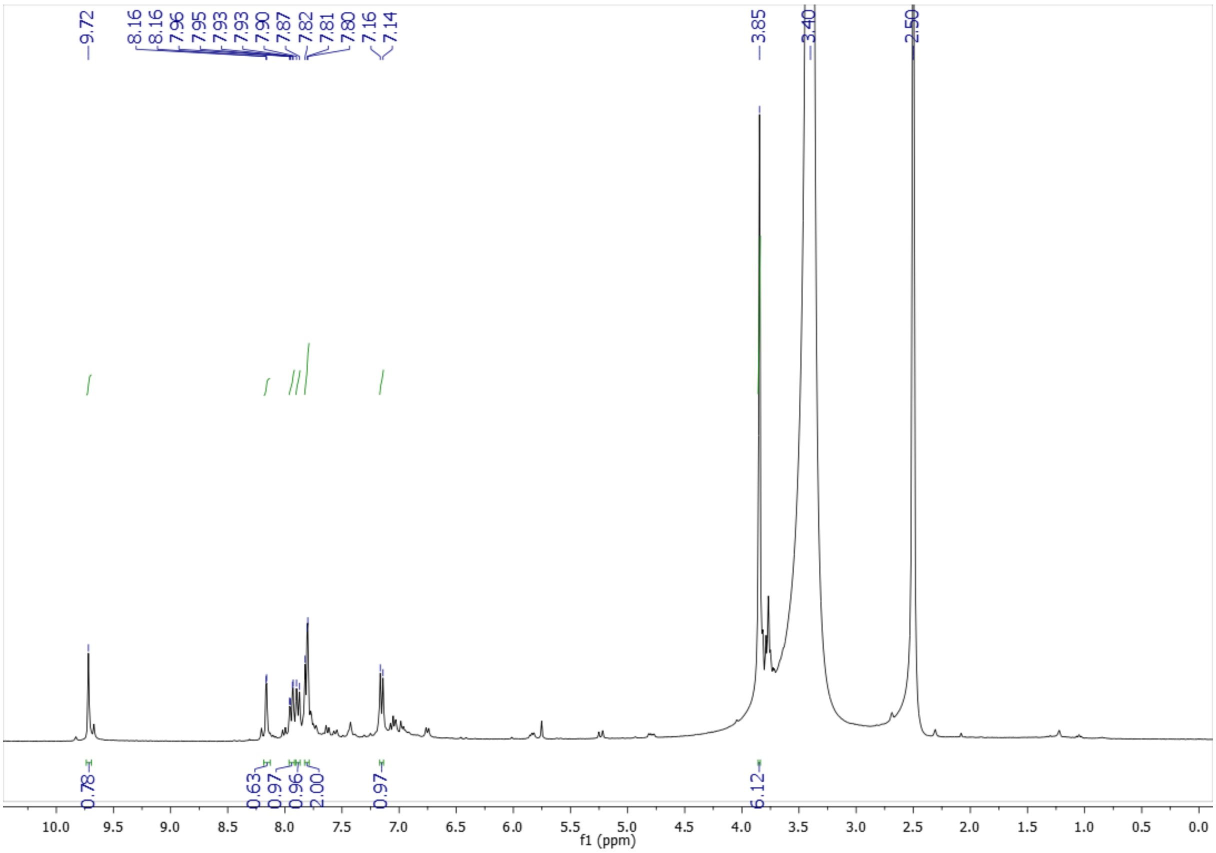

7-Bromo-2-(3,4-dimethoxyphenyl)-3-hydroxy-4H-chromen-4-one (**4b**)

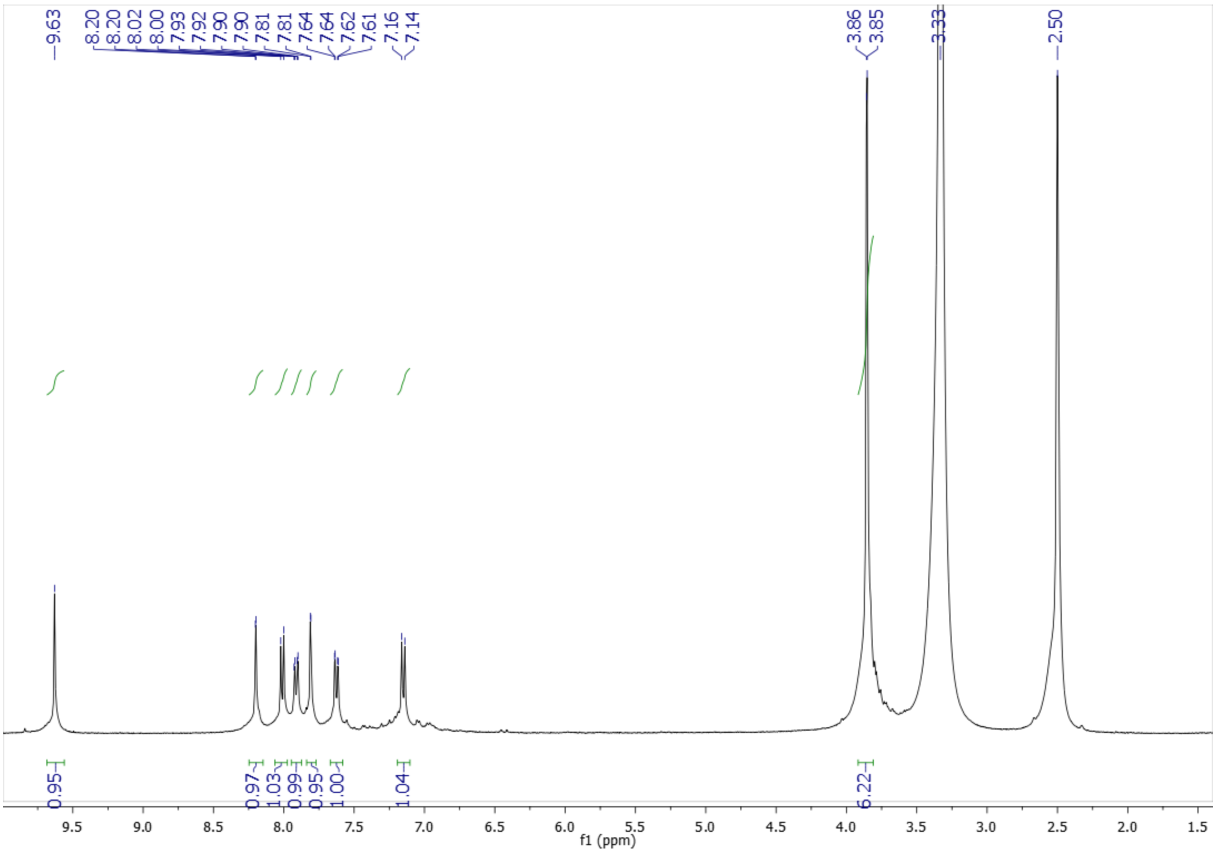

7-Bromo-3-hydroxy-2-(3,4,5-trimethoxyphenyl)-4H-chromen-4-one (**4c**)

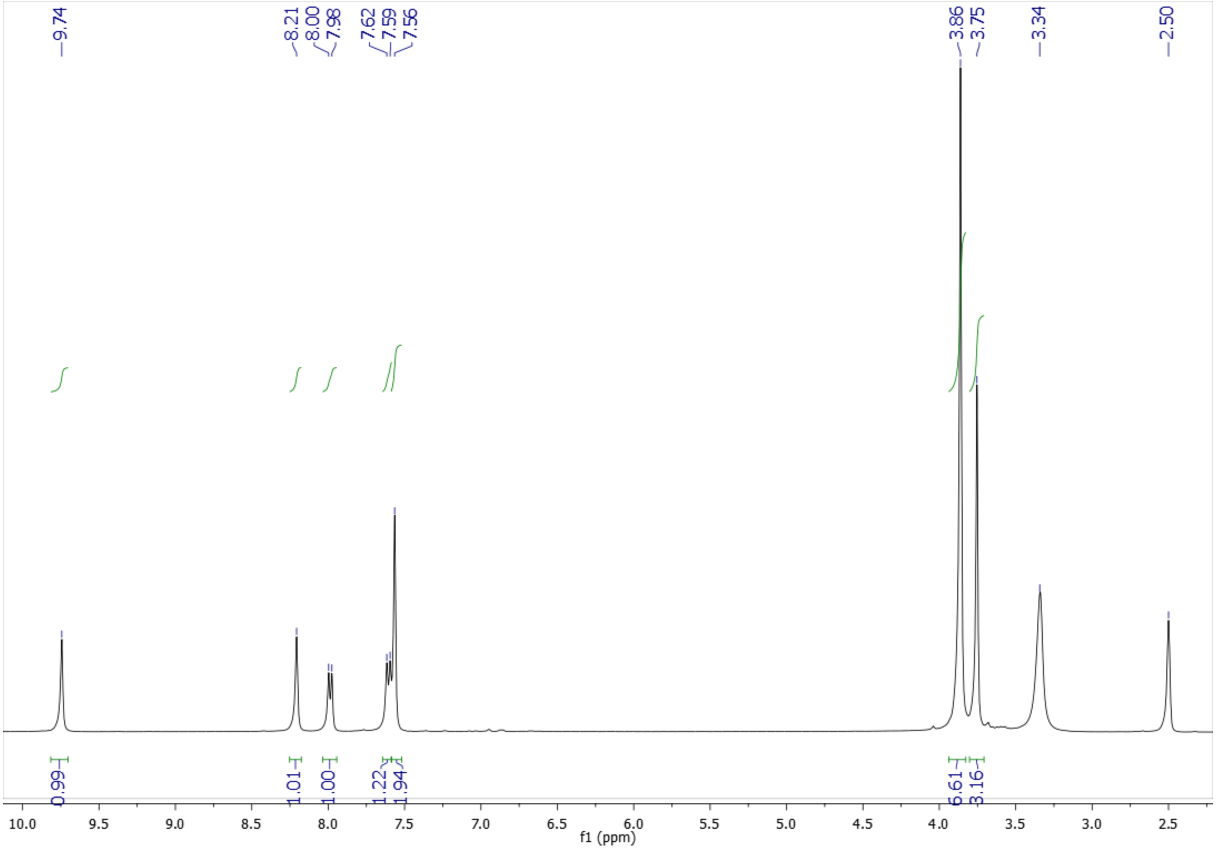

2-(3,4-Dimethoxyphenyl)-6-[3-(dimethylamino)phenyl]-3-hydroxy-4H-chromen-4-one (**6a**)

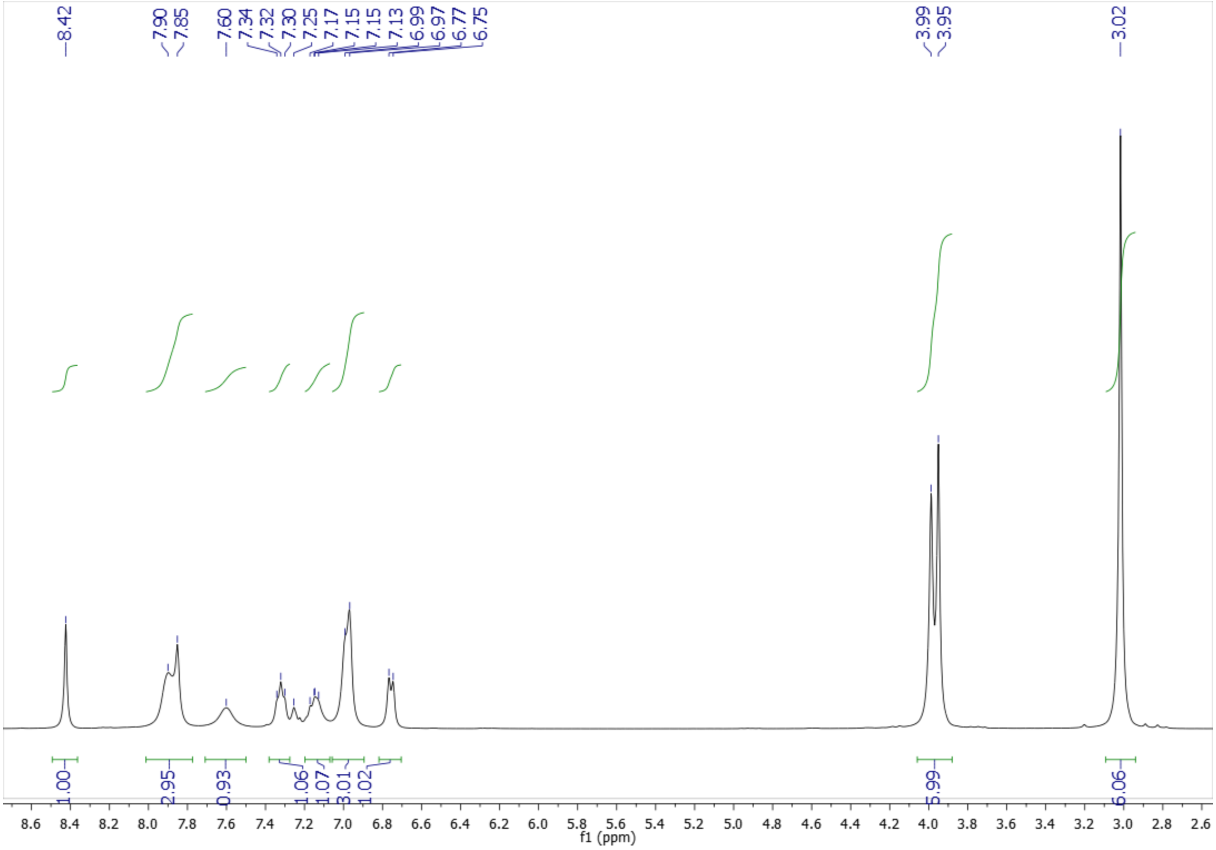

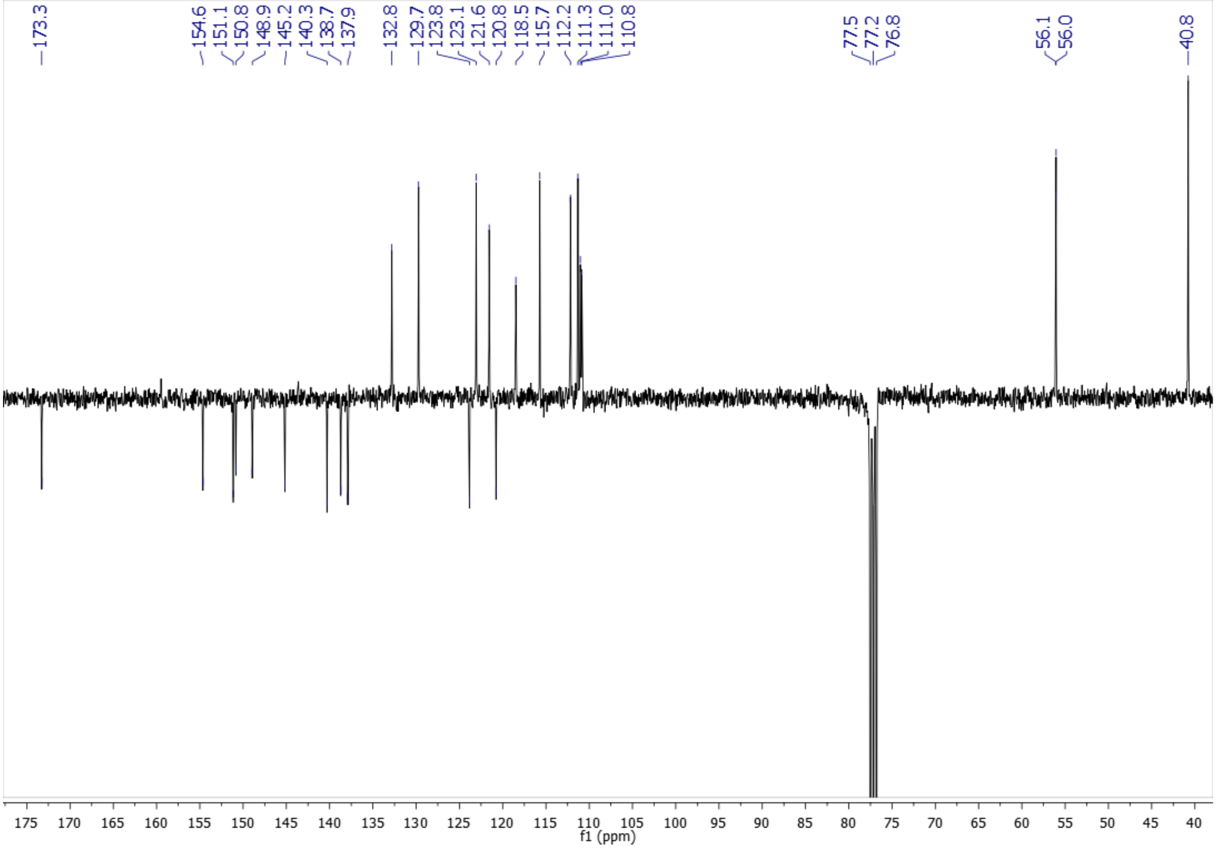

2-(3,4-Dimethoxyphenyl)-3-hydroxy-6-[4-(methoxymethyl)phenyl]-4H-chromen-4-one (**6b**)

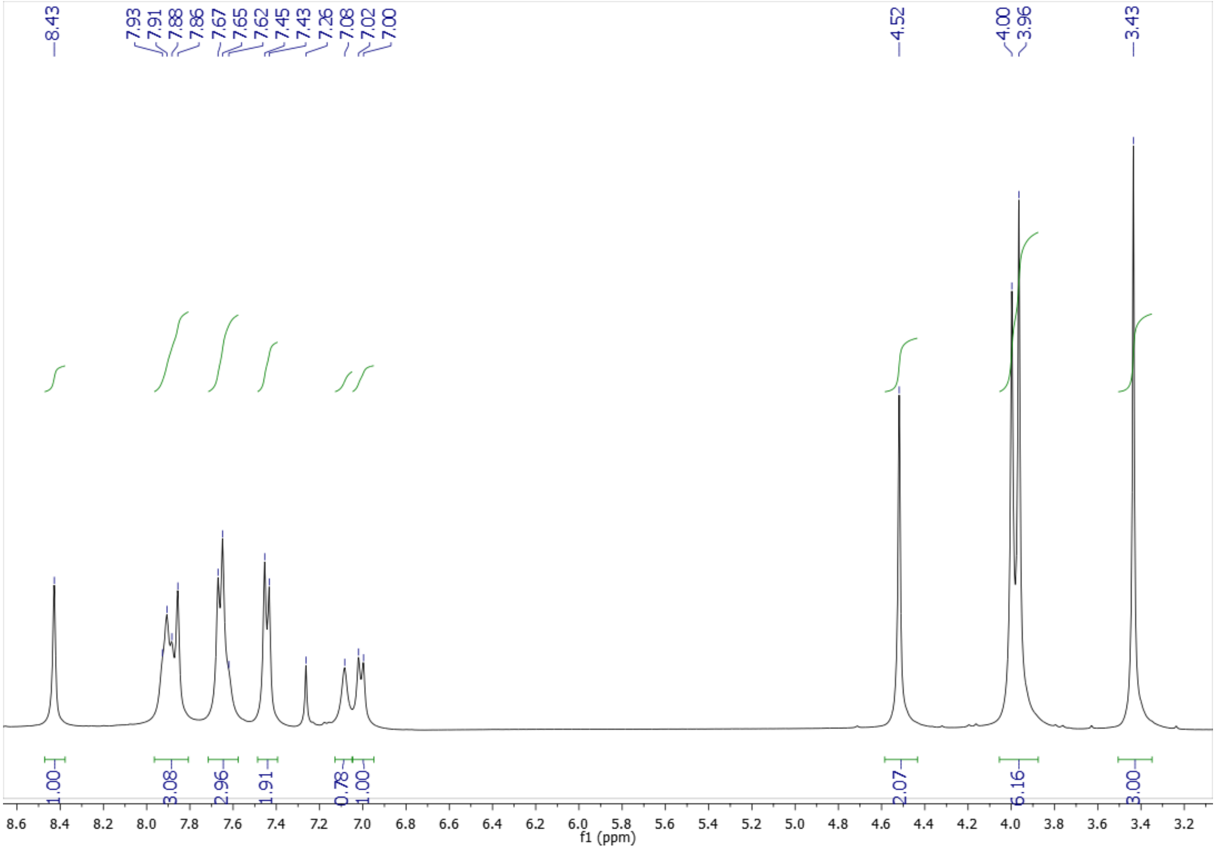

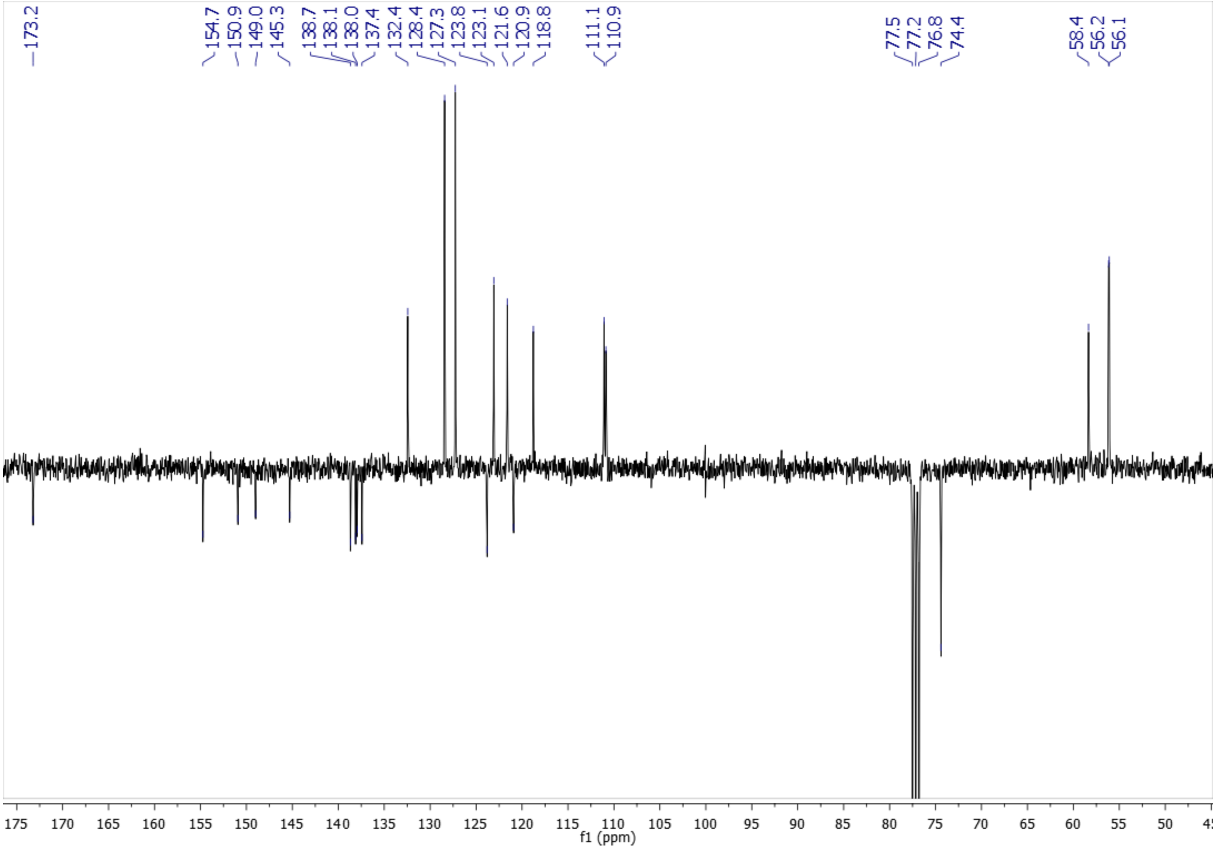

2-(3,4-Dimethoxyphenyl)-7-[3-(dimethylamino)phenyl]-3-hydroxy-4H-chromen-4-one (**6c**)

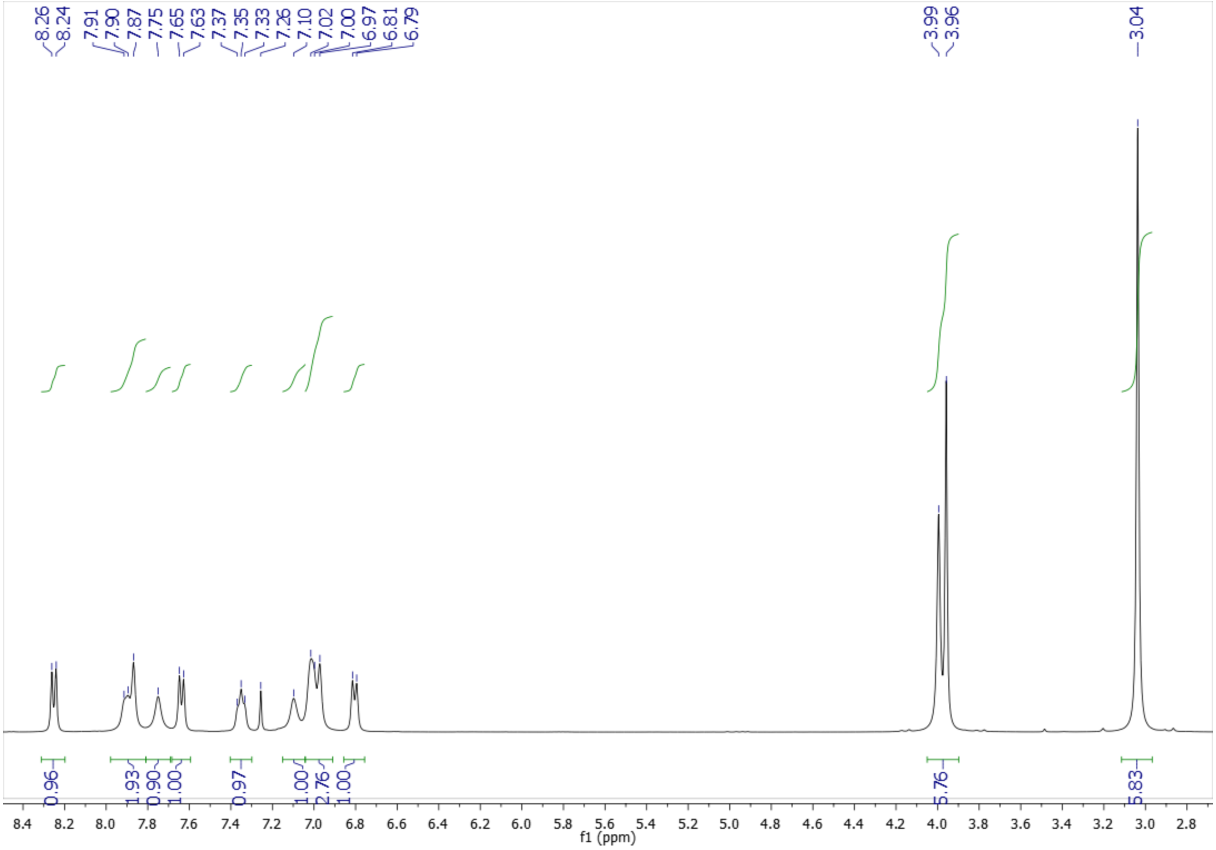

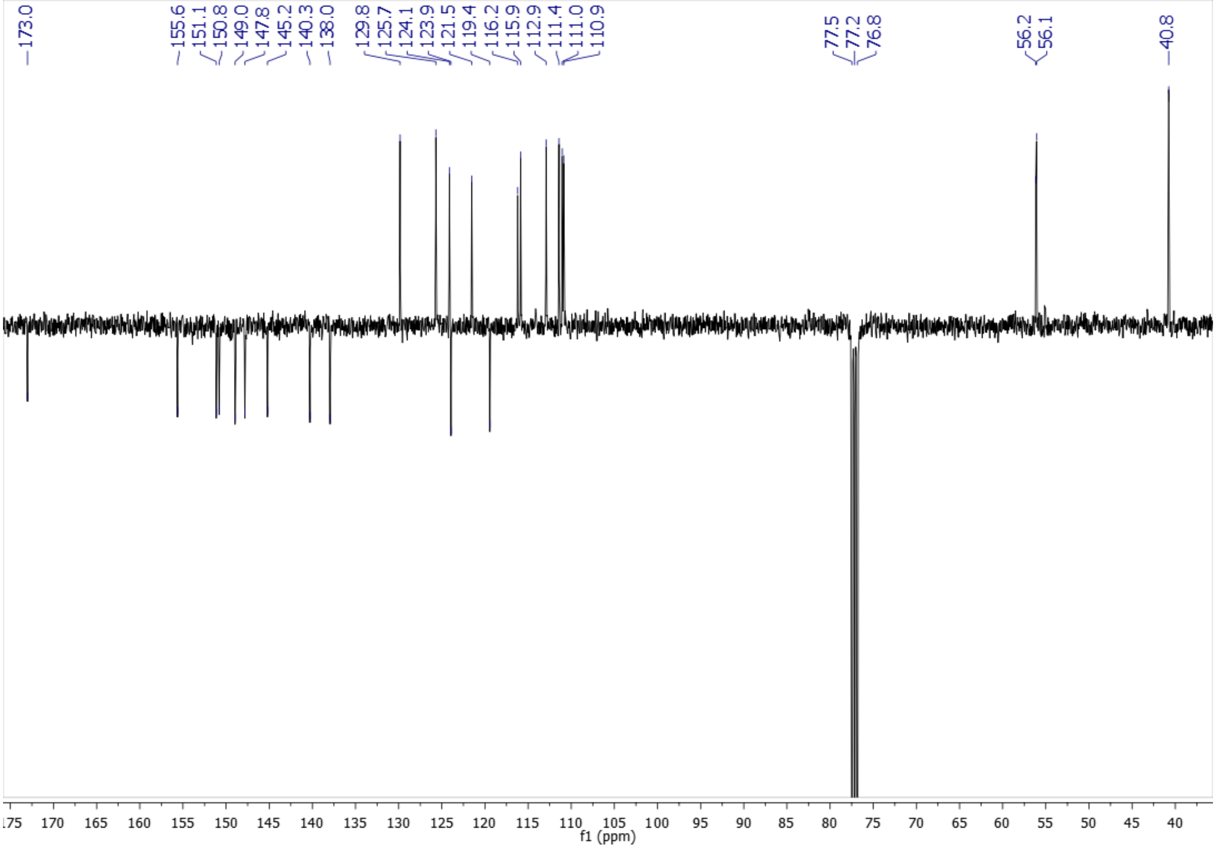

2-(3,4-Dimethoxyphenyl)-3-hydroxy-7-[4-(methoxymethyl)phenyl]-4H-chromen-4-one (**6d**)

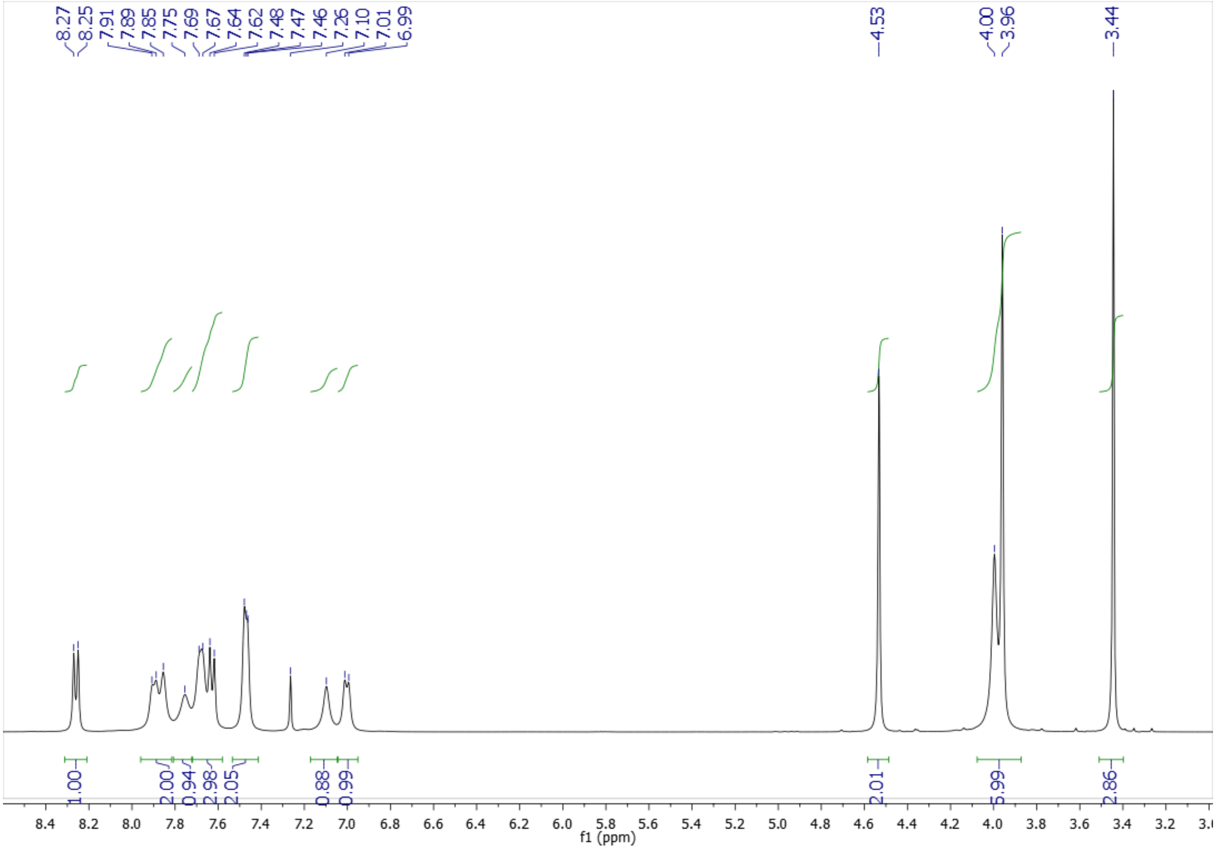

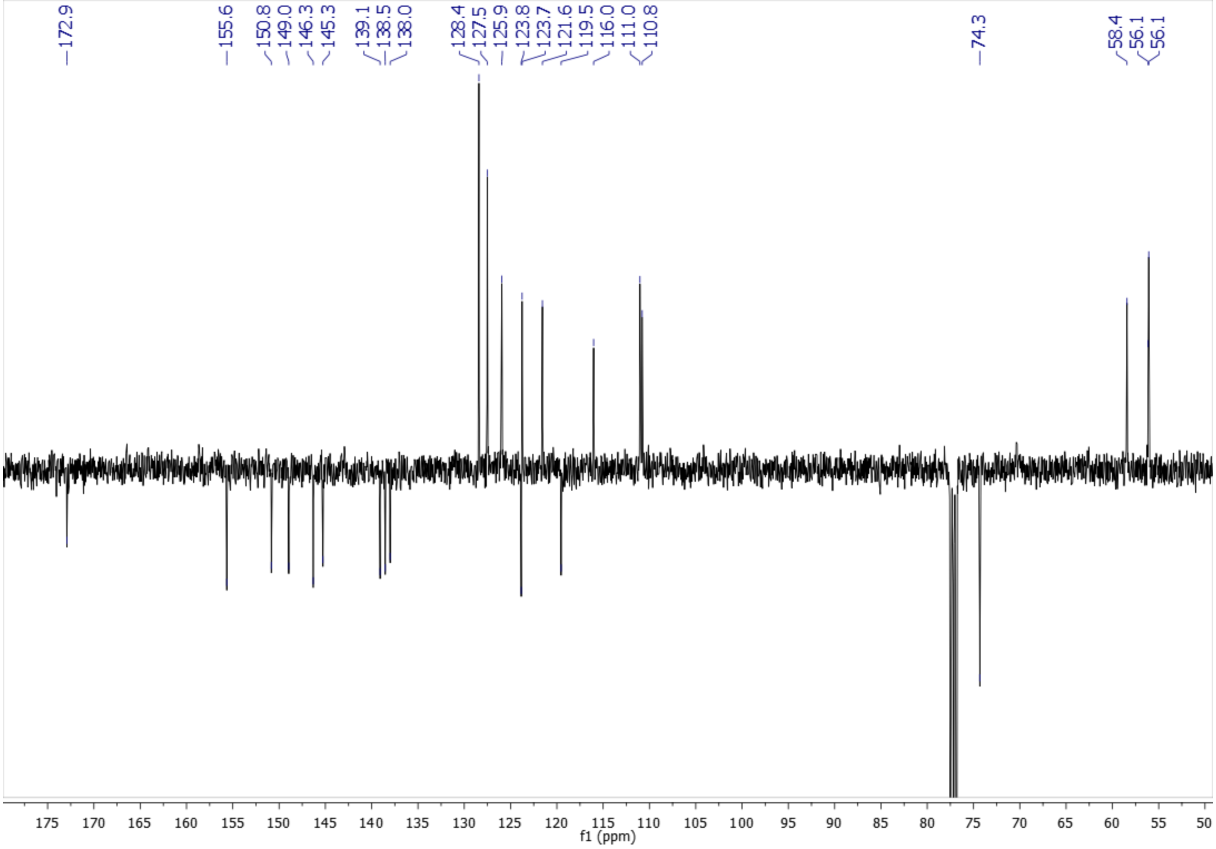

7-[3-(Dimethylamino)phenyl]-3-hydroxy-2-(3,4,5-trimethoxyphenyl)-4H-chromen-4-one (**6e**)

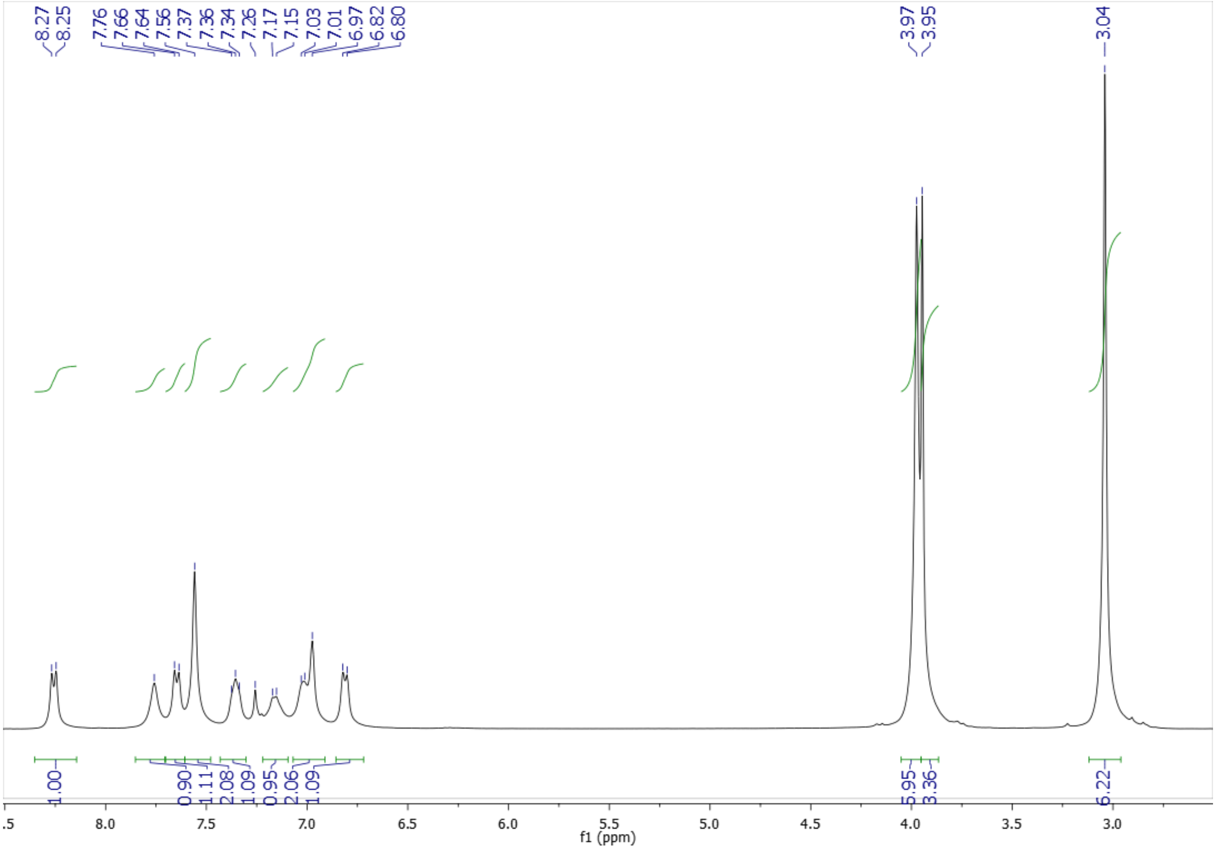

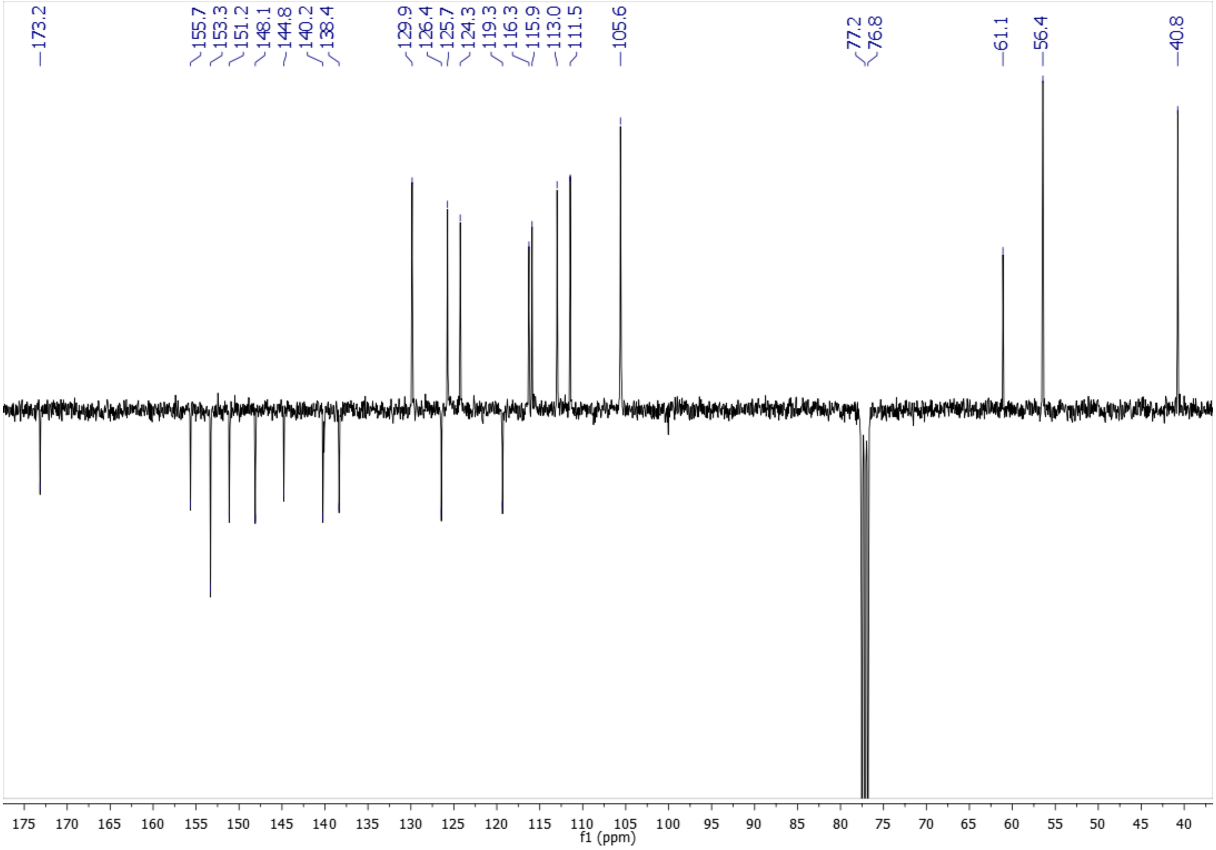

3-Hydroxy-7-[4-(methoxymethyl)phenyl]-2-(3,4,5-trimethoxyphenyl)-4H-chromen-4-one (**6f)**

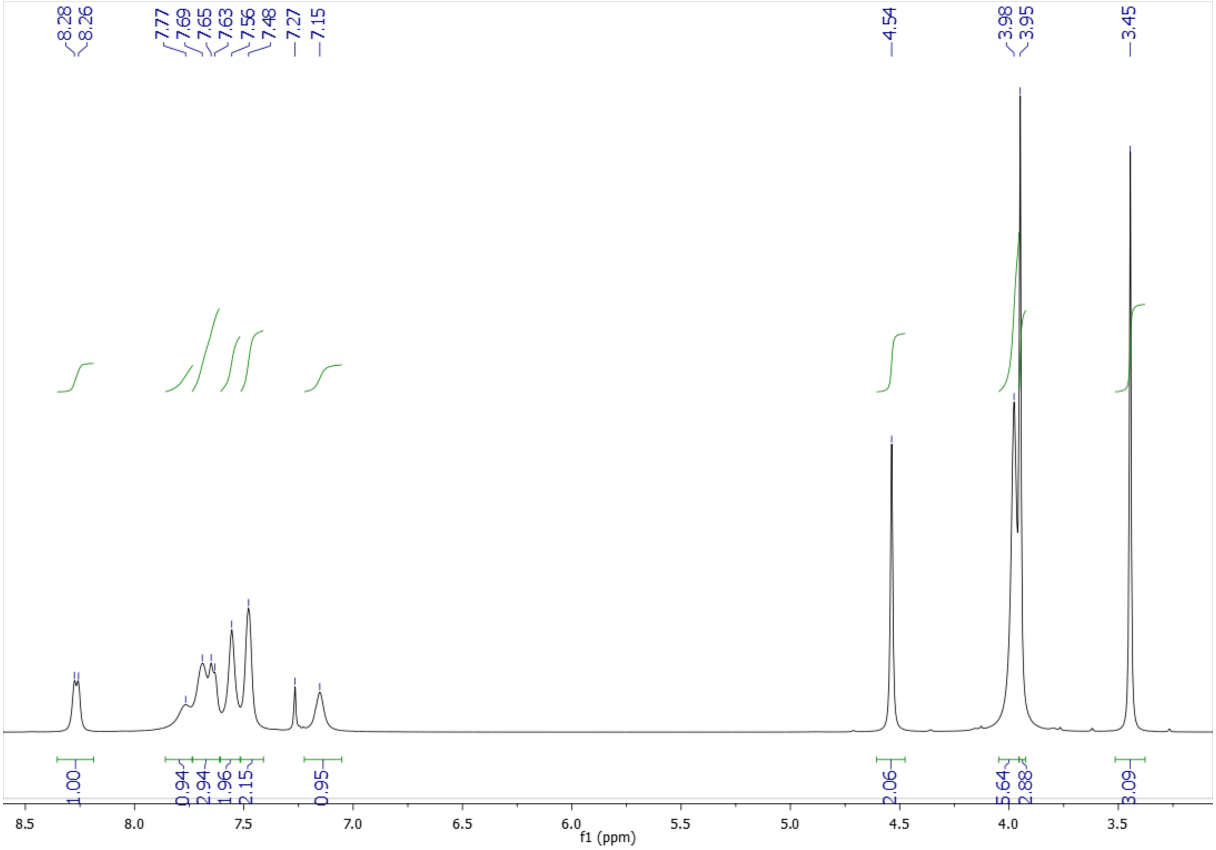

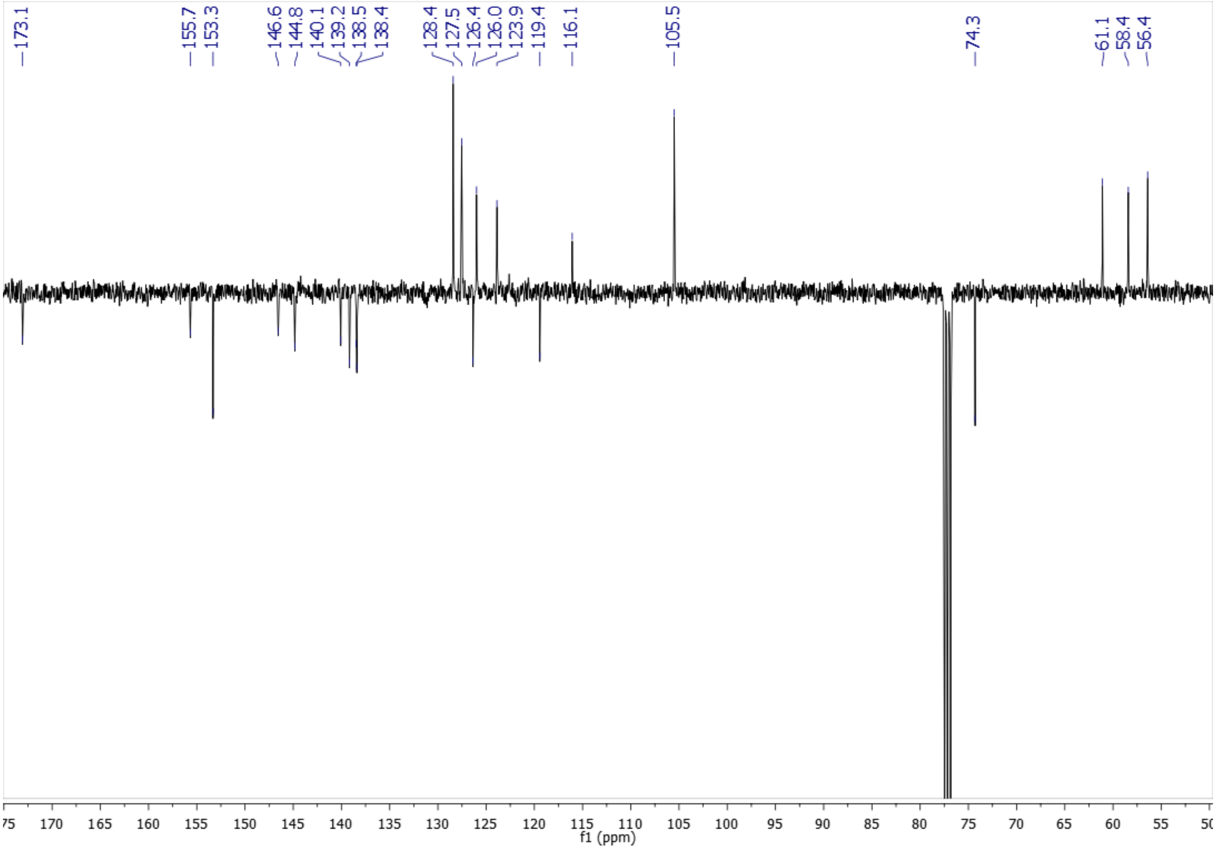

Supplement: Supplementary file 1 [file molecules-23-03161-s001.pdf]
